# LLT1 overexpression renders allogeneic-NK resistance and facilitates the generation of enhanced universal CAR-T cells

**DOI:** 10.1186/s13046-025-03273-2

**Published:** 2025-01-25

**Authors:** Shuxian Zhu, Shiyu Zuo, Chuo Li, Xingjie You, Erlie Jiang, Xiaoming Feng, Yuechen Luo

**Affiliations:** 1 State Key Laboratory of Experimental Hematology, National Clinical Research Center for Blood Diseases, Haihe Laboratory of Cell Ecosystem, Institute of Hematology & Blood Diseases Hospital, Chinese Academy of Medical Sciences & Peking Union Medical College, Tianjin, 300020 China; 2 Tianjin Institutes of Health Science, Tianjin, 301600 China; 3 Geriatric Medical Center, Division of Geriatric Gastroenterology, The First Affiliated Hospital of Kunming Medical University, Kunming, 650032 China; 4 T-Cell Precision Therapy Lab, Department of Pathology and Pathophysiology, School of Basic Medical Sciences, Hangzhou Normal University, Hangzhou, 311121 China; 5 Zhejiang Key Laboratory of Medical Epigenetics, Hangzhou, 311121 China

**Keywords:** Universal CAR-T Cells, NK Cell Inhibition, Allogeneic Rejection, T Cell Stemness

## Abstract

**Background:**

The benefit of universal CAR-T cells over autologous CAR-T cell therapy is that they are a treatment that is ready to use. However, the prevention of graft-versus-host disease (GVHD) and host-versus-graft reaction (HVGR) remains challenging. Deleting class I of human leukocyte antigen (HLA-I) and class II of human leukocyte antigen (HLA-II) can prevent rejection by allogeneic T cells; however, natural killer (NK) cell rejection due to the loss of self-recognition remains unresolved. This study tested whether the overexpression of Lectin-like transcript 1 (LLT1), an NK cell inhibitory ligand, in T cell receptor (TCR) and HLA-I/II disrupted universal CD38-targeting CAR-T cells could prevent rejection by allogeneic NK cells.

**Methods:**

We generated CD38-targeting universal CAR-T cells by transducing T cells with lentiviruses encoding the CD38 CAR and LLT1 constructs. T cells were subjected to CD38, TCR, HLA-I, and HLA-II gene knockdown using CRISPR/Cas9, followed by lentiviral transduction. We performed cytotoxicity, proliferation, and cytokine assays to evaluate the functionality of universal chimeric antigen receptor-T cell (UCAR-T) cells and conducted in vitro and in vivo assays, including allogeneic responses and RNA sequencing, to assess their resistance to allogeneic T and NK cells, anti-leukemia efficacy, and persistence in treating hematologic malignancies.

**Results:**

Genetic editing of CD38 universal CAR-T cells, including CD38, T cell receptor alpha constant (TRAC), beta-2-microglobulin (B2M), and class II major histocompatibility complex transactivator (CIITA) knockdowns, was successfully achieved. In vitro, LLT1 overexpression boosted CAR-T cell proliferation and antitumor activity, leading to a transcriptional signature characterized by elevated stemness-related markers (*SELL*, *BCL6*, *TCF7*, and *CD27*) and increased levels of IL-10 and other cytokines. It also effectively mitigates rejection by allogeneic NK and T cells. In a humanized T-cell acute lymphoblastic leukemia (T-ALL) model, CD38 allogeneic universal CAR-T cells demonstrated superior survival rates and tumor clearance with reduced inflammatory responses.

**Conclusion:**

According to these results, LLT1 overexpression enhances UCAR-T cell activity and prevents allogeneic rejection, providing essential insights for the development of universal CAR-T cell therapy.

**Supplementary Information:**

The online version contains supplementary material available at 10.1186/s13046-025-03273-2.

## Background

Treatment of cancer has been revolutionized by chimeric antigen receptor (CAR)-T cell therapy [1, 2], especially with the development of CD19 CAR-T cells for B cell acute lymphoblastic leukemia and B cell lymphoma [2, 3]. Although autologous CAR-T cells are currently the preferred treatment for most patients, they face several challenges, including high production costs, lengthy manufacturing periods, and decreased cell fitness and functionality caused by prior exposure to disease and chemotherapy, such as suboptimal CD8^+^ /CD4^ +^ ratios and low central memory T-cell (TCM) ratios [4–6]. Additionally, the use of autologous T cells in T-ALL presents a unique risk of inadvertent transduction of leukemic cells, which can compromise safety. Allogeneic universal CAR-T cells, which are currently under clinical trials or in early-stage research, offering a promising solution to these challenges. They can expedite treatment, reduce production costs, and maintain high-quality donor T-cells. Research has shown that TCR knockdown in universal CAR-T cells can effectively mitigate GvHD in recipients [7, 8]. Moreover, T cells derived from healthy donors typically exhibit superior fitness and a more robust response, offering a safer and potentially more effective therapeutic alternative compared to autologous approaches. However, combining TCR knockdown with host lymphocyte depletion via alemtuzumab may lead to pancytopenia and an increased risk of severe infection. To address HLA-related HVGR, researchers have explored the ablation of HLA-I/II through knockdown of B2M and CIITA genes. Nonetheless, this approach may leave CAR-T cells vulnerable to immune rejection by NK cells due to the loss of self-recognition through HLA-I molecules [9, 10].

Overexpression of HLA-E [11] or HLA-G [12]or CD52 knockdown [13]has been reported to mitigate the allogeneic NK response against universal CAR-T cells. However, HLA-E and HLA-G have limitations in preventing rejection by allogeneic NK cells, as they do not simultaneously inhibit all NKG2A^+^ and KIR2DL^+^ NK cell subpopulations. Lectin-like transcript 1 (LLT1), a member of the NK receptor C-type lectin family, was identified as a CD161 ligand in 2005 [14, 15]. LLT1-CD161 and Ly49-HLA-I share strikingly similar expression patterns and play crucial roles in the NK cell self-recognition system. However, it remains unclear whether modulation of LLT1 expression influences the rejection of universal CAR-T cells by allogeneic NK cells.

CD38 is a promising tumor antigen with significant potential for the treatment of multiple myeloma [16]. Its widespread expression has been demonstrated in non-Hodgkin lymphoma (NHL) cells, acute lymphoblastic leukemia (ALL), and acute myeloid leukemia (AML) [17, 18]. Clinical case reports have highlighted the therapeutic potential of CD38-targeted CAR-T cells in ALL and AML [19, 20]. In the therapy of immune thrombocytopenia, novel humanized CD38 monoclonal antibodies (mAbs) that target plasma cells have recently demonstrated encouraging outcomes [21]. We believe that CD38 represents a valuable, broadly applicable target, and that a universal CD38-targeting CAR construct holds promise for treating various hematologic malignancies.

In this study, we present an allogeneic CD38-targeting universal CAR-T cell therapy with low immunogenicity and high allogeneic persistence. These CD38 UCAR-T cells feature CD38 disruption to prevent fratricide, TCR disruption to minimize GVHD, and HLA-I/II disruption to prevent rejection by allogeneic T cells. Importantly, LLT1 was specifically overexpressed to reduce the allogeneic NK cell response. Both in vitro and in humanized mice models, we assessed the immunogenicity, antitumor efficacy, and persistence of UCAR-T cells. Additionally, we explored the potential mechanisms by which LLT1 influences UCAR-T cell function and behavior.

## Methods

### Production of lentivirus

The CD38 CAR was constructed by fusing the CD38 single-chain fragment variable (ScFv) derived from the daratumumab mAb with the CD8 hinge and transmembrane domains, 4-1BB costimulatory domain, and CD3z intracellular domain. Co-expression of CD38 CAR and LLT1 was achieved using the P2A peptide, which links the coding sequence (CDS) of LLT1 isoform 2 downstream of CD3z. CD38 and CD38-LLT1 CAR were cloned into a PCDH lentiviral vector driven by the EF1a promoter. The control plasmid was an empty PCDH vector. 293 T cells were transfected with a polyethyleneimine (PEI; Yeasen, 40816) complex, which included PEI, Rev, VSVG, PMDL, and the plasmid. 48 h after transfection, the supernatant was collected, filtered, and centrifuged for three hours at 5000 g. The resultant viral pellet was kept in sterile EP tubes at minus 80 °C after being reconstituted in either PBS or serum-free culture media.

### UCAR-T manufacturing process

T-cells were isolated from peripheral blood using CD3-positive selection microbeads (STEMCELL, 17851). The isolated T cells were activated by incubation them with CD3/28 activator (STEMCELL, 10971) in Opti-vitro T cell medium (ExCellBio, TE000-N082) with 100 IU/ml of IL-2 (T&L Biotechnology, GMP-TL906) and 10% FBS (ExCellBio, FSP500) added for 2 days. Cas9 protein (Thermo Fisher Scientific, A50577) and sgRNA (UBIGENE, Red Cotton™) were pre-incubated for at least 45 min to form ribonucleoprotein (RNP) complexes. Activated T cells were resuspended in the RNP mixture and electroporated using the Lonza 4D-Nucleofector system. Post-transfection, the T cells were transferred to pre-warmed complete medium. After 24 h, the transfected T cells were exposed to the lentivirus at a multiplicity of infection (MOI) of 10 and centrifuged at 2000 rpm for 1.5 h. Following a 4-days expansion period, T cells with target gene knockdown and positive CAR expression were sorted. The following sgRNA targeting sequences were used:

- TRAC sequence : 5’-ACCCGGCCACTTTCAGGAGG-3’,
- B2M sequence : 5’-TTCCTGAATTGCTATGTGTC-3’,
- CIITA sequence : 5’-GCAGTTGATGGTGTCTGTGT-3’,
- CD38 sequence : 5’-GCGCTTTCCCGAGACCGTCC-3’.

### Cell lines and culture conditions

All cell lines used in this study, including CCRF-CEM, Raji, Molm13, and HEK-293 T, were obtained from the Cell Resource Center of the Institute of Hematology and Blood Diseases Hospital, Tianjin, China. The cell lines were maintained in RPMI-1640 medium (Gibco, 11875500) supplemented with 10% FBS (Excell, FSP500) and 1% penicillin–streptomycin solution (Gibco, 10378016). 293 T cells were maintained with Dulbecco's Modified Eagle Medium (Gibco, 11995065), supplemented with 10% FBS and 1% penicillin–streptomycin solution. To avoid primed peripheral blood mononuclear cells (PBMCs) rejecting CCRF-CEM cells because of an HLA mismatch, which could impact tumor growth, the B2M gene was knocked out in the CCRF-CEM cell line. After infection with the PCDH-luciferase-GFP virus and genome editing with the Cas9 SNP, single-cell flow sorting (FACS Aria II; BD Biosciences) was employed to isolate the CCRF-CEM-Luc-GFP-B2M^KO^, CCRF-CEM-Luc-GFP, Raji-Luc-GFP, and Molm13-Luc-GFP cell lines. Mycoplasma contamination was tested monthly using mycoplasma detection kits (Yeasen, 40601), and short tandem repeat (STR) analysis was used three times per year to validate the cell lines.

### Cytotoxicity assay in vitro

CCRF-CEM-Luc-GFP, Raji-Luc-GFP, and Molm13-Luc-GFP tumor cells were cultured in appropriate medium until they reached logarithmic phase. CAR-T and PCDH-T cells were cultured for no longer than 14 days. After counting viable cells with trypan blue (Gibco, 15250061) staining, effector and target cells were seeded in 96-well plates at various E:T ratios and incubated for 12 h. Negative control wells contained only target cells without effector cells. DAPI dye (Yeasen, 40728) was diluted to 1% in PBS (Servicebio, G4202) and added to the cells. To accurately count the remaining tumor cells, 10 µL of precision count beads (BioLegend, 424902), at a concentration of 1 × 10^5^/ml, were added to each well and mixed. Flow cytometry data were collected by analyzing 20,000 beads per sample. The proportion of DAPI^−^GFP^+^ cells was used to quantify the remaining tumor cells. The Specific Cytolysis effect targeting tumor cells was calculated using the following formula: Specific Cytolysis = ( Number of cells in negative control- Number of remaining tumor cells)/Number of cells in negative control × 100%

### Proliferation assay in vitro

CD38 CAR-T cells (1 × 10^5^/mL) with various gene knockouts were cultured in 24-well plates in T cell medium containing FBS and IL-2, without additional stimuli. Cell proliferation was monitored every three days by counting cells using flow cytometry with precision count beads.

### CFSE assay

To evaluate T cell proliferation in response to antigen stimulation, CAR-T cells were incubated with CFSE (Invitrogen, C34554), a fluorescent dye that enters cells and labels them. As cells divide, the fluorescence intensity decreases, generating a series of peaks in the flow cytometry histogram. T cell proliferation was assessed by measuring CFSE fluorescence intensity in individual cells using flow cytometry.

### Cytokine measurements

For cytokine assays, 1 × 10^5^ sorted CAR-T cells were co-cultured with 1 × 10^5^ CCRF-CEM-Luc-GFP cells in 24-well plates, in 1 ml of medium, for 24 h. Supernatants were collected and analyzed for human cytokines and chemokines using the LEGENDplex Human Panel (BioLegend, 741189). Data analysis was performed using the online LEGENDplex Qognit software.

### Allogeneic primary NK assay

Ficoll-Paque (LDS1075) was used to layer peripheral blood, and density gradient centrifugation was used to separate peripheral blood mononuclear cells (PBMCs). CD3-negative PBMCs were obtained by isolating CD3-positive cells using CD3 microbeads. The CD3-negative PBMCs were then cultured in X-vivo 15medium (Lonza, 02-053Q) supplemented with 5% human AB serum (Sigma, H4522), 10 ng/ml IL-15 (PeproTech, 200-15) and 100 IU/ml IL-2 for 14 days to induce NK cell expansion. CD3^-^CD56^+^ NK cells were successfully expanded to a purity of up to 50-90%. Subsequently, NK cells were enriched using CD56 magnetic beads (Miltenyi Biotec, 130-097-042) through positive selection.

Target CAR-T cells were stained with a violet cell-tracking dye (BioLegend, 425101) to distinguish them from NK cells during flow cytometry. Target and effector NK cells were co-cultured at E:T ratios of 2:1, 1:1, 1:2, and 1:4 for 24 h. The number of remaining target cells was counted, and specific lysis o was calculated by the formula: % Specific CAR-T Cell Lysis = (Number of Control CAR-T cells-Number of Remaining CAR-T Cells) / Number of Control CAR-T cells × 100%.

For 48 h, four-fold CCRF-CEM-Luc-GFP cells and one-fold allogeneic NK cells were co-cultured with violet-dyed CAR-T cells. The control group contained NK and CCRF-CEM cells in a 1:4 ratio. Beads were counted to calculate the number of remaining CAR-T cells and GFP-positive tumor cells. Specific Cytolysis was calculated using the formula: Specific cytolysis = (Number of GFP^+^ cells in control groups-Number of GFP^+^ cells in Samples) / Number of GFP + cells in control groups × 100%. Additionally, the expansion of CAR-T cells was determined using the following formula: Expansion Fold change = Number of cells on Day 2 / Number of cells on Day 0.

### Mixed lymphocyte reaction

CAR-T cells were subjected to 25 Gy irradiation to halt their proliferation and serve as stimulators in the co-culture. PBMCs from allogeneic donors were cultured with the irradiated stimulator cells at 4:1 ratio in X-vivo 15medium (Lonza, 02-053Q) supplemented with 10% FBS and 100 IU/ml IL-2 for 14 days. Following priming, PBMCs were stained with antibodies specific for T cells, B cells, NK cells, DC, and macrophages. Primed PBMCs were labeled with a cell-tracing dye (BioLegend, 425101) and co-cultured with either irradiated or unirradiated allogeneic CAR-T cells for 48 h at a 4:1 ratio. The percentage of CD107a^+^ cells in the primed PBMCs was quantified by flow cytometry. Additionally, CAR-T cells were stained with Annexin V (Biyotime Biotechnology, C1062) and 7-AAD (BD, 559925) to assess apoptosis. The proportion of CAR-T cells killed by the primed PBMCs was determined by bead counting.

### Elisa assays

Supernatants from co-cultures of allogeneic reactive PBMCs and irradiated CAR-T cells were stored at -80 °C. Following a fivefold dilution, each sample was analyzed using the Human IFN-γ Precoated ELISA Kit (Reddot Biotech, 1110002). Data were processed according to established protocols and algorithms, with all results falling within the calibration curve.

### Mouse ALL model

Female NSG mice (6–10 weeks old; NOD *Prkdc*^*cscid*^* Il2rg*^*tm1*^/Bcgen) were purchased from BiocytoGen (Beijing, China). Each NSG mouse was engrafted with 3 × 10^5^ CCRF-CEM-Luc-GFP cells via intravenous injection. To minimize bias, mice were randomly assigned to receive different CAR-T/PCDH-T cells after tumor implantation. Five or seven days later, 2.5 × 10^6^ CAR-T cells were administered intravenously. Tumor burden was monitored weekly using the Xenogen IVIS-200 bioluminescence imaging system starting from Day 0. Every week, a hematology analyzer was used to measure the number of cells in the blood that was drawn from each mouse's tail vein, diluted seven times, and examined. The proportion of CAR-T cells in the blood was determined by flow cytometry, and the number of CAR-T cells per 100 μl of blood was calculated from the cell count data. Following the labeling of extracellular markers, cells were permeabilized and fixated for intracellular staining of cytokines and chemokines, such as CCR2, IL-10, and IL-4. Each experimental group consisted of five mice to ensure adequate statistical power for detecting significant differences. This experiment was repeated independently two to three times.

### Humanized Mouse ALL model with alloreactive PBMCs

NSG mice were intravenously inoculated with 5 × 10^5^ CCRF-CEM-Luc-GFP-B2M^KO^ cells and 5 × 10^6^ HLA-A2 mismatched alloreactive PBMCs, primed as described above. Three or five days later, 2.5 × 10^6^ CAR-T cells and 2.5 × 10^6^ NK cells were injected intravenously at a 1:1 ratio. Tumor progression, CAR-T cell numbers, and CAR-T cell phenotype were monitored as described previously. To evaluate the effect of rechallenged NK cells, the same dose of NK cells was re-injected seven days after the initial CAR-T cell infusion. Mice were sacrificed based on predefined humane endpoints, including stooped posture, significant weight loss, or mobility difficulties. Bone marrow cells were extracted after sacrifice, and the proportion of tumor and CAR-T cells was determined by flow cytometry. Additionally, the liver, spleen, kidney, brain, skin, and other organs were preserved in 4% paraformaldehyde, sectioned, and stained with hematoxylin and eosin (H&E). These sections were examined under a microscope at 100 × and 200 × magnification.

### RNA-seq and analysis

After 48 h of coculturing with the specified cells, CAR-T cells were sorted using flow cytometry (FACS). RNA sequencing was performed on the DNASEQ-T7 platform (BGI-Shenzhen), generating 150 base paired-end reads. Heat maps were generated based on DEGs filtered by [Log2FC] > 1 and FDR p-value < 0.05, with DEG heat maps constructed using TBtools (RRID:SCR_023018). Gene Set Enrichment Analysis (GSEA) was carried out using the software GSEA_4.3.3 provided (Broad Institute), utilizing gene sets from The Broad Institute Molecular Signature Database, including:

- GSE26928_NAIVE_VS_CENT_MEMORY_CD4_TCELL_UP
- GSE9650_NAIVE_VS_MEMORY_CD8_TCELL_DN
- KEGG_CYTOKINE_CYTOKINE_RECEPTOR_INTERACTION
- GOBP_T_CELL_CYTOKINE_PRODUCTION
- GOBP_CANONICAL_NF_KAPPAB_SIGNAL_TRANSDUCTION

### Intracellular cytokine staining

CAR-T/PCDH-T cells were co-cultured with CCRF-CEM cells in a 1:1 ratio. The co-culture was treated with a combination of PMA/ionomycin mixture (250 ×) (Multi Sciences, CS1001) and GolgiStop (BD Biosciences, 554724) for 6 h. Tumor cell surfaces were labeled with an anti-human CD38 antibody. Live/dead cell discrimination was performed using FVD (eBioscience, 65-0865-14). The cells were then fixed and permeabilized using BD IntraSure™ Kit (BD Bioscience, 641776). Intracellular staining for human IL-10, was performed by incubating the cells with an anti-human IL-10 antibody (BioLegend, 501421) for 30-60 min, followed by washing with wash buffer. The staining procedure for TCF7 was performed as described above, except that tumor cells and PMA stimulation were not included.

### Phosphorylation staining

CAR-T cells were pre-treated with JSH-23 (MCE, HY-13982) for 12 h. 5 × 10^4^CAR-T cells were co-cultured with 5 × 10^4^ CCRF-CEM cells in the presence of CD38 antibody for 30 min. Following co-culture, cells were incubated with FVD (eBioscience, 65-0865-14) for live/dead discrimination. After that, the cells were fixed for 20 min at room temperature in 4% paraformaldehyde. To get rid of extra fixative, cells were rinsed with PBS containing 1% BSA after fixation. To permeabilize the cells, they were incubated with 90% (v/v) ice-cold methanol for 20 min. After permeabilization, cells were washed thoroughly with PBS to remove residual methanol. Next, the cells were incubated with a phospho-NF-kB p65 (Ser536) (Cell Signaling Technology, 5733S) for 30–60 min. Following antibody incubation, the cells were rinsed with PBS and their phosphorylation levels were measured by flow cytometry.

### Statistical analysis

GraphPad Prism Version 10.2.3 (RRID:SCR_002798) was used for data analysis and graph creation. Based on at least three separate trials, the data are shown as the mean ± SEM. As applicable, the log-rank test (Mantel-Cox), unpaired Student's t-test (two-tailed), or one-way ANOVA were used to determine statistical significance. The following symbols in figure legends indicate statistical significance: * *P* < 0.05, ** *P* < 0.01, *** *P* < 0.001, and **** *P* < 0.0001.

## Results

### Multiple gene editing to generate CD38 UCAR-T cells

**Fig. 1:**
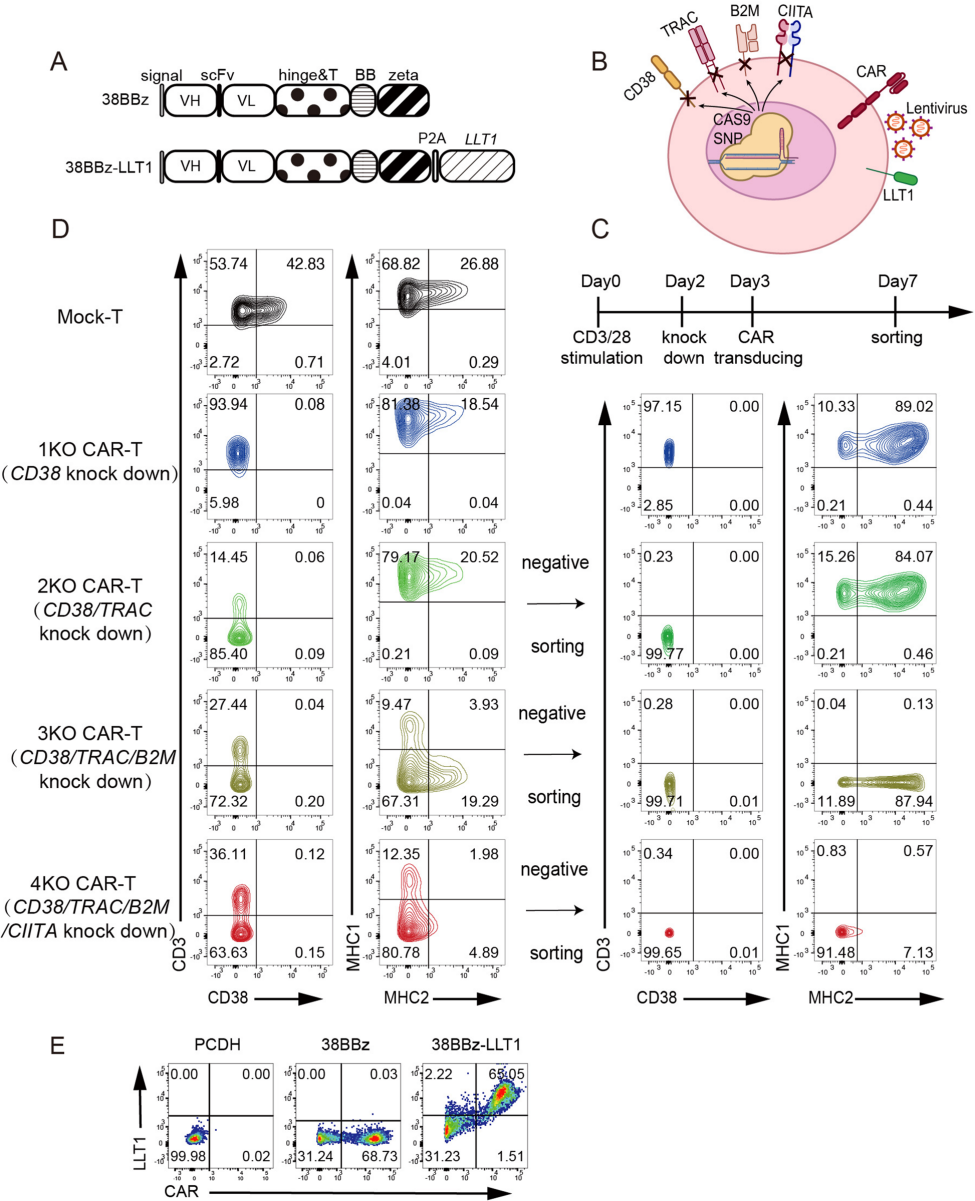
Multiple gene editing to generate CD38 UCAR-T. **A** Schematic representation of CD38 CAR and LLT1-containing CD38 CAR constructs. The CD38 CAR construct comprises the CD8 signal peptide (signal), the VH and VL domains (ScFv), the hinge and transmembrane domains of CD8 (hinge & T), the 4-1BB costimulatory domain (BB), and the CD3z intracellular domain(zeta). The LLT1 coding sequence is ligated to the CD3z domain, forming the CD38-LLT1 CAR containing LLT1. **B** Schematics of the genome editing approach design. **C** Schedule and methods for production of universal CD38 CAR-T. **D** The results of flow plots demonstrating the knockdown of T-cell surface markers when SgRNAs targeting *TRAC*, *CD38*, *B2M*, and *CIITA* were used in combination, as well as negative sorting to obtain 1KO, 2KO, 3KO, and 4KO CAR-Ts. **E** Flow plots showing the efficiency of CD38 CAR and LLT1 overexpression in CAR-T cells

The anti-CD38 CAR vector was constructed by incorporating the CD8a signal peptide, the ScFv sequence of daratumumab, the 4-1BB costimulatory domain, and the CD3z intracellular signaling domain (Fig. 1A). However, as previously reported, 38BBz CAR-T cells exhibited limited proliferation and underwent apoptosis (Fig.S1A and B), probably because of CD38 expression in activated T cells [22]. To prevent this, we created CD38 knockout (KO) CD38 CAR-T (referred to 1KO CAR-T cells) by designing an sgRNA targeting the CD38 gene, which enabled CAR-T cells to continue expanding (Fig.S1B).

Additionally, the *TRAC*, *B2M*, *CIITA*, and *CD38* genes in primary human T cells were disrupted using the CRISPR/Cas9 technology to prevent GVHD [23]. Cas9-mediated knockdown and lentiviral-mediated overexpression were achieved simultaneously and efficiently, as outlined in Fig. 1B and 1C. This approach demonstrated that electroporation with a combination of multiple sgRNAs achieved 50% knockout efficiency compared to mock T cells electroporated without sgRNA. The viability and apoptotic rate of CAR-T cells were unaffected by the simultaneous knockdown of the four genes, as shown by DAPI, 7-AAD, and Annexin V staining (Fig.S1A and C), compared to the knockdown of a single gene. For comparison of the effects of *TRAC*, *B2M*, and *CIITA* knockdown on CAR-T cell immunogenicity, we classified CAR-T cells into 0KO (38BBz CAR-T), 1KO, 2KO (CD38/TRAC KO), 3KO (CD38/TRAC/HLA-I KO), and 4KO (CD38/TRAC/HLA-I/HLA-II KO) groups (Fig. 1D). Although HLA molecules are essential for allogeneic immunological rejection, their absence can allow allogeneic CAR-T cells to be recognized and attacked by NK cells [9].

We used P2A ligation to clone CD38 CAR and LLT1 sequences into lentiviral PCDH vectors in order to investigate whether LLT1 overexpression in HLA-deficient CAR-T cells may decrease allogeneic NK cell rejection [24](Fig. 1A). Primary T cells from healthy donors were isolated and transfected to express both CD38 CAR and LLT1 (Fig. 1E). Disruption of *CD38*, *TRAC*, *B2M* and *CIITA*, combined with lentiviral transduction, generated anti-CD38 4KO-LLT1 CAR-T cells.

### Overexpression of LLT1 preserve naive/stem phenotype of CAR-T cells

**Fig. 2:**
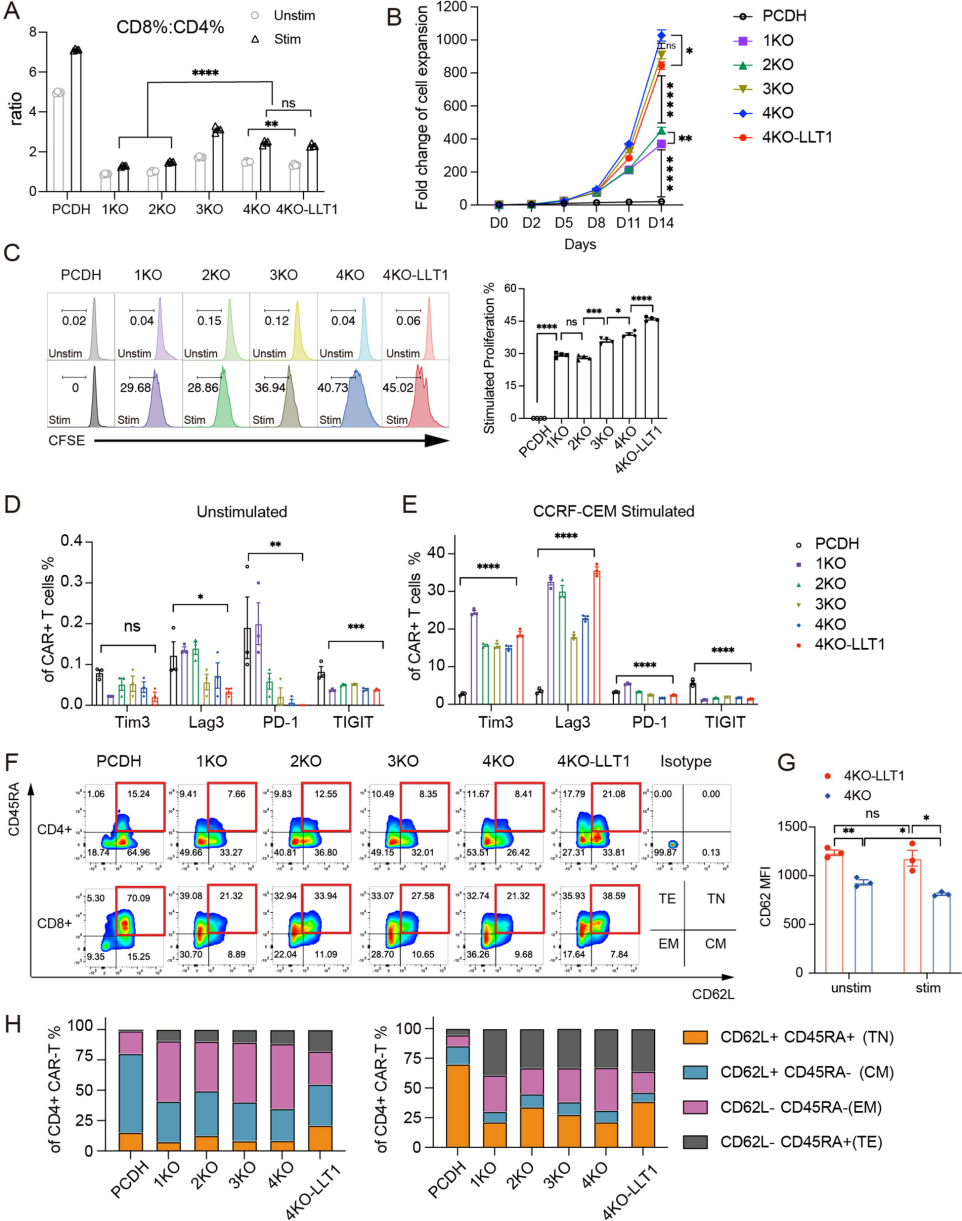
Overexpression of LLT1 preserve naive/stem phenotype of CAR-T cells. **A** The ratio of the proportion of CD8^+^ T cells to the proportion of CD4^+^ T cells in unstimulated and antigen-stimulated condition (*n* = 4). **B** The fold plots illustrate the fold expansion of CAR-T/PCDH-T cells in T-cell medium with 100 ng/ml IL-2 and 10%FBS within 14 days. **C** CFSE assay exhibited the proliferation of CAR-T/PCDH-T cells in unstimulated and antigen-stimulated condition for 72 h. The right diagram shows the level of CAR-T proliferation in response to antigenic stimulation (*n* = 4). **D** and **E** The results show the surface expression of immune checkpoint molecules on CAR-T/PCDH-T cells. Statistical significance was determined with one-way ANOVA. **D** The expression of PD-1, LAG3, and TIM-3 was evaluated in unstimulated condition (*n* = 3). **E** The expression of PD-1, LAG3, and TIM-3 was evaluated in antigen-stimulated condition (*n* = 3). **F** Flow plots show the memory phenotype of CAR-T cells, TN, naive, CD62L^+^CD45RA^+^; CM, central memory, CD62L^+^CD45RA^−^; EM, effector memory, CD62L^−^CD45RA^−^; TE, terminal effector, CD62L^−^CD45RA^+^. **G** The histogram depicts the difference in CD62L MFI levels between 4KO CAR-T and 4KO-LLT1 CAR-T cells after 24 h of CCRF-CEM stimulation, as assessed by flow cytometry (*n* = 3). **H** The histogram shows frequency of memory phenotypic proportions of CAR-T cells. The data presented here are the mean ± SEM of three independent experiments. *p < 0.05, **p < 0.01, ***p < 0.001, and ****p < 0.0001; ns, not significant

Preliminary experiments indicated an increase in the CD8/CD4 ratio (Fig. 2A) and a two-fold increase in the expansion capacity of CAR-T cells without tumor stimulation (Fig. 2B) when B2M and CIITA were knocked down. The ability of CAR-T cells to expand in the presence of target antigens is crucial for eradicating tumor burden and maintaining durable remission [25]. Accordingly, we evaluated the in vitro proliferative capacity and marker expression in CAR-T cells following antigen stimulation. The 3KO and 4KO CAR-T cells exhibited enhanced proliferation (Fig. 2C) and demonstrated lower expression levels of PD-1, Lag3, and TIM3 compared to 1KO CAR-T cells (Fig. 2E). The reduced expression of immune checkpoint molecules may be attributed to the absence of MHC class I and II molecules [26], which limit the involvement of the TCR pathway, thereby reducing T-cell exhaustion signaling. Furthermore, LLT1 overexpression did not alter the CD8/CD4 ratio upon antigen stimulation (Fig. 2A), but it significantly preserved the population of CD38 CAR-T cells exhibiting a naive T cell phenotype (Fig. 2F). After 24 h of antigen stimulation, the 4KO-LLT1 group showed a higher mean fluorescence intensity (MFI) of CD62L compared to the 4KO group (Fig. 2G). Clinical studies suggest that lymphocytes with naive (TN) and stem-like cell memory (TSCM) phenotypes are associated with better therapeutic responses due to their proliferative capacity and longer survival time [6]. A significant proportion of the 4KO-LLT1 CAR-T cells were CD62L^+^CD45RA^+^ naive T cells (Fig. 2H). Furthermore, upon stimulation with CCRF-CEM cells, the 4KO-LLT1 CAR-T cells exhibited higher levels of PD-1, TIM3, and Lag3 and lower levels of TIGIT than the 4KO CAR-T cells (Fig. 2E). They also showed enhanced proliferation upon antigen stimulation (Fig. 2C).

### LLT1 overexpression promoted in vitro cytotoxicity and in vivo anti-tumor activity

**Fig. 3:**
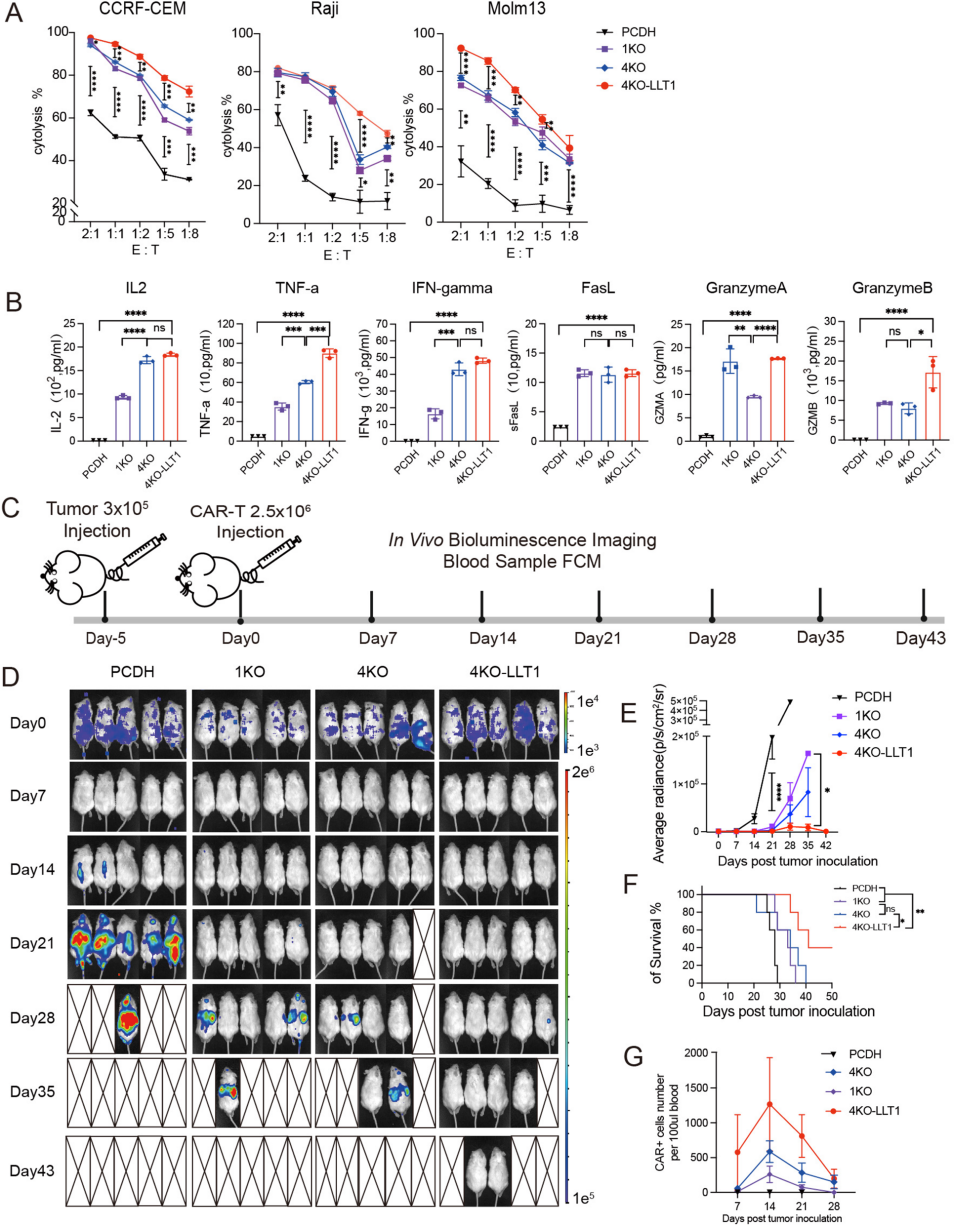
LLT1 overexpression promoted in vitro cytotoxicity and in vivo anti-tumor activity. **A** The graph illustrates the cytotoxic activity of CAR-T cells after a 12-h period of co-culture with target cells at a range of E:T ratios (*n* = 3). **B** The concentration of secreted cytokines in the supernatants of CAR-T cells co-cultured with target cells in a 1:1 ratio for 24 h (*n* = 3). **C** Flowchart shows that mice (*n* = 5) were injected intravenously with 3 × 10^5^ CCRF-CEM-Luc-GFP cells, followed by 2.5 × 10^6^ CAR-T/PCDH-T cells five days later. Tumor burden of mice was measured every week. **D** Bioluminescence imaging (BLI) results of tumor burden. Control group refers to PCDH-T cells treated mice. **E** The mean value of average radiance in each group of mice was monitored using bioluminescence in order to compare the therapeutic efficacy of 4KO-LLT1 CAR-T with that of other CAR-T cells. **F** Survival curves of NSG ALL model (*n* = 5), the statistical significance was determined with log-rank test (Mantel-Cox). **G** The plot shows the quantity of CAR-T cells per 100 µl of mouse peripheral blood. Flow cytometry was conducted once a week to identify HuCD45^+^ CAR^+^ cells within the peripheral blood of mice. Data represent the mean ± SEM of triplicates. *p < 0.05, **p < 0.01, ***p < 0.001, and ****p < 0.0001; ns, not significant

We compared CD38 expression levels across a panel of cell lines representing various hematological malignancies and found that the AML cell line Molm13, lymphoma cell lines Nalm6 and Raji, and T-ALL cell lines CCRF-CEM and Molt4 exhibited particularly high CD38 expression (Fig.S3A). The CCRF-CEM, Raji, and Molm13 cell lines were selected as target cells and transfected with lentiviruses to express luciferase (Luc) and green fluorescent protein (GFP) (Fig.S3B). In the 12-h cytotoxicity assay, 4KO CAR-T, 1KO CAR-T, and 4KO-LLT1 CAR-T cells showed potent cytotoxicity against the target cell lines (Fig. 3A). The cytotoxicity of 4KO CAR-T cells against CCRF-CEM cells was comparable to that of 1KO CAR-T cells. TRAC, B2M, and CIITA knockdown did not influence the cytotoxicity of CAR-T cells over a short period. Compared to 4KO and 1KO CAR-T cells, 4KO-LLT1 CAR-T cells demonstrated noticeably greater cytotoxicity against the T-lineage tumor CCRF-CEM, especially at lower effector-to-target (E:T) ratios. We then assessed the cytokine release from CAR-T cells in response to CCRF-CEM cells after a 24-h co-culture at a 1:1 ratio. All three CAR-T cell types produced Fas ligand (FasL), a member of the TNF receptor superfamily, in response to CCRF-CEM cells. Crucially, compared to 1KO CAR-T cells, 4KO CAR-T and 4KO-LLT1 CAR-T cells generated noticeably more interleukin-2 (IL-2), tumor necrosis factor alpha (TNF-α), and interferon-gamma (IFN-γ) (Fig. 3B). The enhanced proliferative expansion capacity observed in 4KO CAR-T cells, as compared to 1KO CAR-T cells, may be attributed to the higher release of IL-2 [27], which may result in the enhanced cell-lysis capability of 4KO CAR-T cells. Additionally, the levels of TNF-α, granzyme A (GZMA), and granzyme B (GZMB) by 4KO-LLT1 CAR-T cells were higher than that observed in 4KO CAR-T. Taken together, our findings indicate that 4KO-LLT1 CAR-T cells exhibit effective and specific cytotoxicity against CD38^+^ tumor cells, with HLA knockdown promoting CAR-T cell proliferation and LLT1 overexpression enhancing their cytotoxicity.

To assess the in vivo efficacy of CAR-T cells, CCRF-CEM-Luc-GFP cells were injected intravenously into the tail veins of NSG mice to establish a T-ALL model. The therapeutic effect of CD38 CAR-T cells on leukemia progression was evaluated on days 5 and 7 post-transplantation (Fig. 3C and S3D). Mice injected with 1KO CAR-T cells showed the earliest recurrence of tumor growth (Fig. 3E and S3F). In contrast, the 4KO-LLT1 CAR-T cell group exhibited the longest remission period among all groups (Fig. 3F and S3G). Flow cytometric analysis of CD45^+^ CAR^+^ cells in 100μl of peripheral blood from mice revealed that on days 14 and 21, 4KO-LLT1 and 4KO CAR-T cells expanded significantly more than 1KO CAR-T cells (Fig. 3G and S3H).

To confirm that the effects of LLT1 overexpression were independent of gene silencing, we conducted in vitro experiments to compare 38BBz-LLT1 CAR-T cells with 38BBz CAR-T cells lacking gene knockouts. According to our findings, LLT1 overexpression enhanced the naive phenotype of CAR-T cells by substantially raising the percentage of CD62L-positive cells (Fig.S4A-B). Furthermore, upon stimulation with tumor antigens, LLT1 overexpression improved the anti-tumor efficacy (Fig.S4C). Notably, LLT1 also reduced CAR-T cell rejection by NK cells and PBMCs (Fig.S4D-F).

### 4KO-LLT1 CAR-T cells exhibit elevated cytokine production and enhanced naive/stemness transcriptional signature

**Fig. 4:**
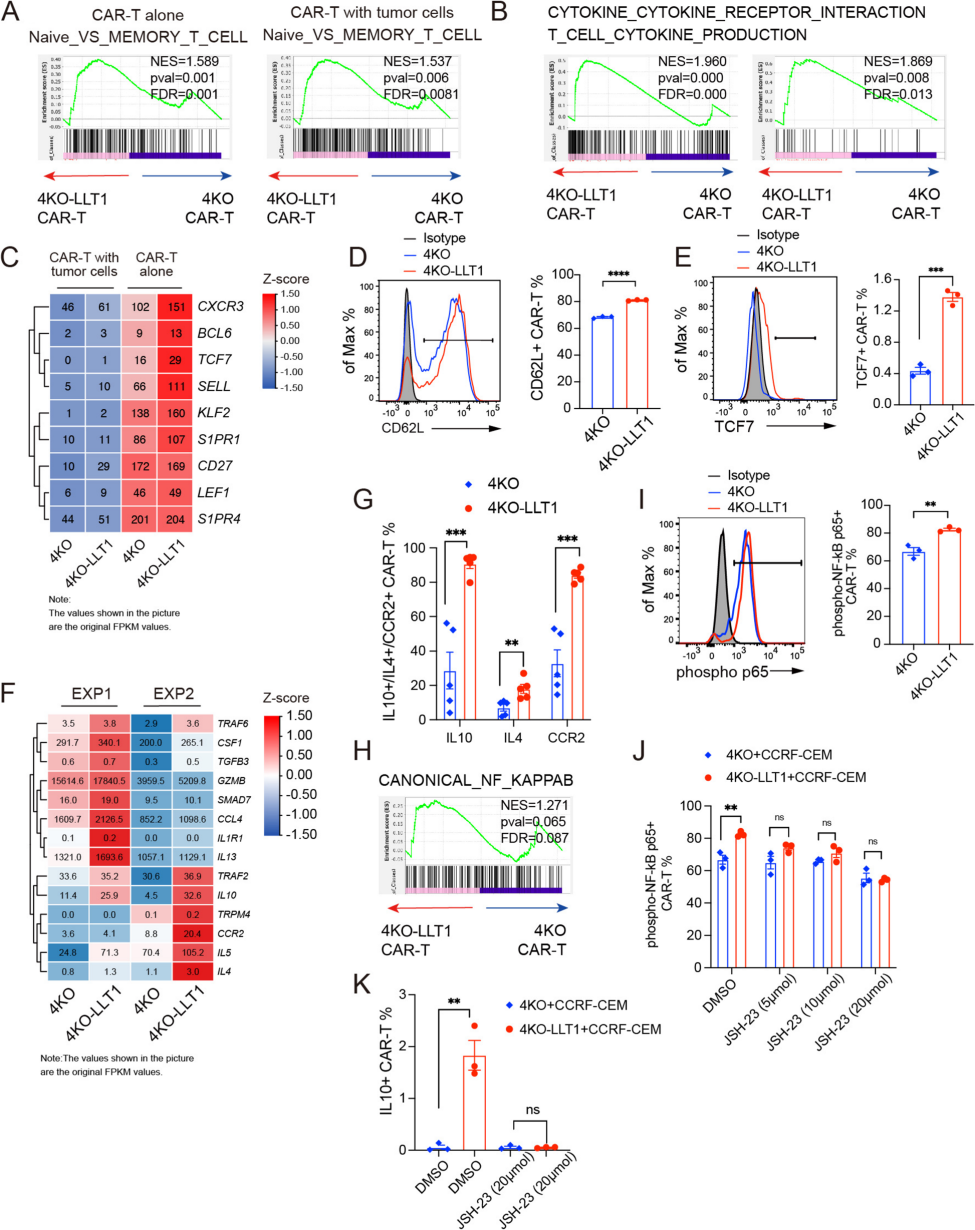
4KO-LLT1 CAR-T cells exhibit elevated cytokine production and enhanced naive/stemness transcriptional signature. **A** Representative GSEA results, demonstrated significantly upregulation of naive-related genes in 4KO-LLT1 CAR-T cells versus 4KO CAR-T cells before and after tumor infiltration. **B** Representative GSEA results, demonstrated significantly upregulation of cytokine-related genes in 4KO-LLT1 CAR-T cells versus 4KO CAR-T cells after tumor infiltration. Each group has two replicates from different donors. **C** The known related stemness-related gene are shown in the heatmap. **D** The expression of CD62L in CAR-T cells (*n* = 3). **E** Intensity of *TCF7* in CAR-T cells (*n* = 3). **F** The known related cytokine-related gene are shown in the heatmap. The RNA sample from two independent replicates of the experiment. **G** Histogram plots show the percentage of *IL10*, *IL4* and *CCR2* expression in CAR-T cells from peripheral blood using flow cytometry. Data represent the mean ± SEM (*n* = 5). **H** Representative GSEA results, demonstrated significantly upregulation of canonical NF-kB signaling pathway in 4KO-LLT1 CAR-T cells versus 4KO CAR-T cells after tumor infiltration. **I** Intensity of phosphor-NF-kB p65 (Ser536) in CCRF-CEM pre-incubated CAR-T cells (*n* = 3). **J** Percentage of phosphor-NF-kB p65 (Ser536) expression in CCRF-CEM pre-incubated CAR-T with 5, 10, 20 µmol JSH-23 (*n* = 3). **K** Percentage of IL-10 expression in CCRF-CEM pre-incubated CAR-T with DMSO and 20 µ mol JSH-23 (*n* = 3). Statistical significance was determined by two-tailed unpaired Student's t-test. *p < 0.05, **p < 0.01, ***p < 0.001, and ****p < 0.0001; ns, not significant

After 48 h of co-culturing with tumor cells, we constructed transcriptome analysis of 4KO-LLT1 and 4KO CAR-T cells to investigate the mechanism by which LLT1 overexpression affects CAR-T cell functionality. The transcriptional profiles of both 4KO-LLT1 and 4KO CAR-T cells were significantly altered upon tumor exposure, with distinct differences observed between 4KO-LLT1 CAR-T and 4KO CAR-T cells (Fig.S5A). Gene Set Enrichment Analysis (GSEA) revealed that 4KO-LLT1 CAR-T cells exhibited positive enrichment in gene signatures associated with naive T cells, both CAR-T cells alone and in the presence of tumor, compared to 4KO CAR-T cells (Fig. 4A). In addition, GSEA analyses demonstrated that LLT1 overexpression led to positive enrichment in pathways related to cytokine and cytokine-receptor interactions, as well as cytokine production (Fig. 4B). In particular, 4KO-LLT1 CAR-T cells exhibited a substantial upregulation of stemness-related genes, including *BCL6*, *TCF7*, *SELL*, and *CD27*, in contrast to 4KO CAR-T cells (Fig. 4C). Following 48 h of tumor stimulation, stemness-related genes were generally downregulated; however, *SELL* and *CD27* expression in 4KO-LLT1 CAR-T cells remained almost twice as high as in 4KO CAR-T cells. Flow cytometric assays further revealed that 4KO-LLT1 CAR-T cells exhibited higher levels of surface CD62L (Fig. 4D), which is consistent with the transcriptomic data for *SELL*. Notably, we observed a significant upregulation of *TCF7*, a key transcription factor that regulates T cell memory [28], in 4KO-LLT1 CAR-T cells. This finding was further validated by intracellular flow cytometry (Fig. 4E), and aligns with previous studies that have highlighted *TCF7*'s critical role in promoting T cell memory. Based on this, we hypothesize that LLT1 maintains the stemness phenotype of CAR-T cells by enhancing TCF7 expression. Correspondingly, the mRNA levels of the cytokine and chemokine were significantly higher in 4KO-LLT1 CAR-T cells stimulated with tumor cells than in 4KO CAR-T cells (Fig. 4F), with *IL-10* showing the most pronounced fold-change. Furthermore, in vivo intracellular staining of CAR-T cells from mice demonstrated elevated *IL-10*, *IL-4* and *CCR2* proteins in the 4KO-LLT1 group (Fig. 4G and S6A).

GSEA also revealed significant enrichment of the canonical NF-kB pathway in the 4KO-LLT1 group (Fig. 4H). To explore this further, we investigated the phosphorylation status of p65 within the NF-kB canonical pathway and observed enhanced activation of this pathway in the 4KO-LLT1 CAR-T cells during tumor antigen presentation (Fig. 4I). Importantly, this pathway activation could be inhibited by the NF-kB nuclear translocation inhibitor JSH-23 [29]. Prior research has shown that during tumor immune responses, NF-kB pathway activation stimulates the release of IL-10 [30]. Our studies revealed that JSH-23 (20 µmol) treatment lowered phosphorylated p65 levels in the 4KO-LLT1 group to levels comparable to the 4KO group (Fig. 4J). Furthermore, IL-10 secretion from the 4KO-LLT1 group was reduced to levels comparable to those observed in the 4KO group (Fig. 4K and S5B). In conclusion, these findings suggest that LLT1 overexpression enhances the naive/stemness phenotype of CAR-T cells and improves their cytokine production, potentially through the canonical NF-kB signaling pathway.

### LLT1 overexpression renders resistance to allogenic NK cell-mediated lysis

**Fig. 5:**
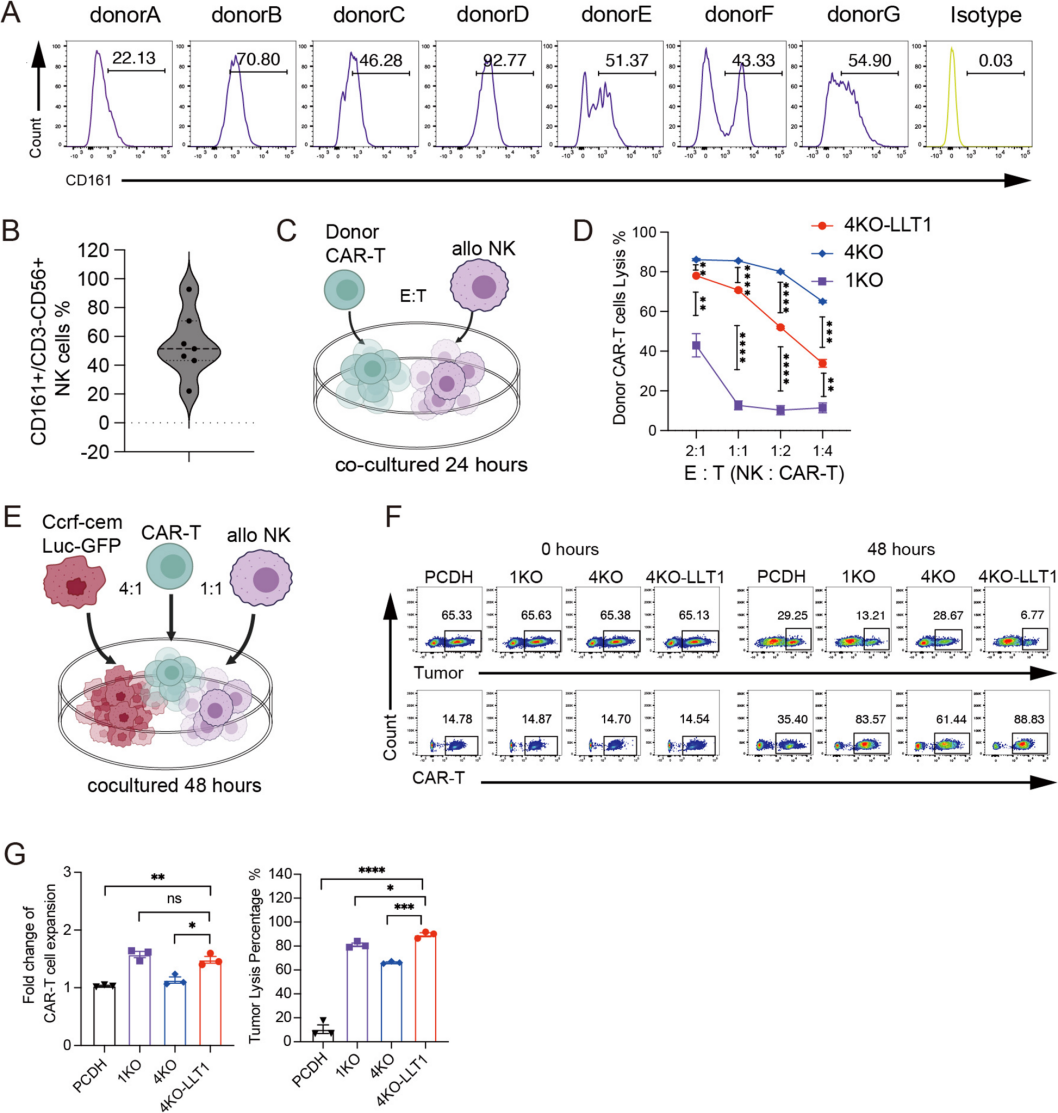
LLT1 overexpression renders resistance to allogenic NK cell-mediated lysis. **A** The flow plots illustrate the proportion of CD161 marker expression on the surface of NK cells in the peripheral blood of seven healthy donors. **B** The violin plot illustrates the distribution of data on the proportion of CD161 in (A) (*n* = 7). **C** The schematic illustration of a model in which autologous primary NK cells eliminate donor UCAR-T cells. **D** The plots present the proportion of lysed UCAR-T cells (*n* = 3) when autologous primary NK cells and donor UCAR-T cells were co-cultured at different E:T ratios over a 24-h period. **E** Schematic illustration of the model simulating the in vivo lysis of tumor cells by CAR-T cells and NK cells. The donor CAR-T/PCDH-T cells were mixed and cultured with onefold allogeneic NK cells and fourfold CCRF-CEM-Luc-GFP cells for 48 h. **F** Representative flow plots showing the percentage of CAR-T cells and tumor cells in the well plate at the two time points of day 0 and day 2. **G** The left diagram shows the ratio of CAR-T cells number compared to the initial CAR-T cells number following a 48-h reaction period, as assessed by flow cytometry counting (*n* = 3). This ratio represents the level of CAR-T cells expansion. The right diagram depicts the ratio of GFP-positive tumor cells lysed after 48 h of reaction in comparison to the control group, which comprised NK cells and tumor cells without CAR-T cells (*n* = 3). Data represent the mean ± SEM of triplicates. Statistical significance was determined by two-tailed unpaired Student's t-test. *p < 0.05, **p < 0.01, ***p < 0.001, and ****p < 0.0001; ns, not significant

The inhibitory NKR-P1A receptor (CD161) and its ligand LLT1 form an MHC-independent recognition system and are genetically related to the NK gene complex-encoded type II transmembrane C-lectin-associated proteins [31]. The CD161 expression levels on the NK cell surface were measured using peripheral blood samples from seven healthy persons (Fig. 5A and S7A), with the average expression exceeding 50% (Fig. 5B). In order to evaluate the protective impact of LLT1 overexpression, CAR-T cells and purified CD3^-^CD56 ^+^ primary NK cells were co-cultured for 24 h at different E : T ratios (Fig. 5C). The results demonstrated that LLT1 overexpression significantly protected the CAR-T cells from NK cell-mediated lysis (Fig. 5D). Additional experiments with NK cells from different donors confirmed the consistency of this protective effect, despite variations in CD161 expression and cytotoxic capacity (Fig.S7B-C). For instance, NK cells from one donor (Fig.S7B), which exhibited around 20% CD161 expression, showed consistent results reinforcing this protective trend. After 72 h of co-culturing NK and CAR-T cells at a 1:4 ratio, 4KO-LLT1 CAR-T cells lysed at the same rate as 1KO CAR-T cells, but at a much lower rate than 4KO CAR-T cells (Fig.S7D). Furthermore, the percentage of surface CD107a, a marker of NK cell degranulation, was significantly reduced in NK cells co-cultured with LLT1-overexpressing CAR-T cells (Fig.S7E).

In order to test the anti-tumor effectiveness and resistance to NK cell rejection for universal CAR-T treatments, we simulated the allogeneic setting of CAR-T therapy by evaluating the tumor-killing capacity of 4KO-LLT1 CAR-T cells with attack of NK cells. Four-fold CCRF-CEM-Luc-GFP cells and one-fold allogeneic NK cells were co-cultured with one-fold violet-dyed CAR-T cells (Fig. 5E). At 0 and 48 h, the proportion and quantity of CAR-T and tumor cells were measured by flow cytometry, with tumor cells and NK cells acting as controls. At 48 h, the proportions of 4KO-LLT1 and 1KO CAR-T cells were significantly higher than those of 4KO CAR-T and PCDH-T cells (Fig. 5F). Notably, the fold expansion of 4KO-LLT1 and 1KO CAR-T cells was similar with both showing superior tumor-killing capacities compared to 4KO CAR-T and PCDH-T cells (Fig. 5G). The 4KO-LLT1 CAR-T cells demonstrated even greater potency that the 1KO CAR-T cells (Fig. 5G).

### LLT1 overexpression renders resistance to allogenic PBMCs

**Fig. 6:**
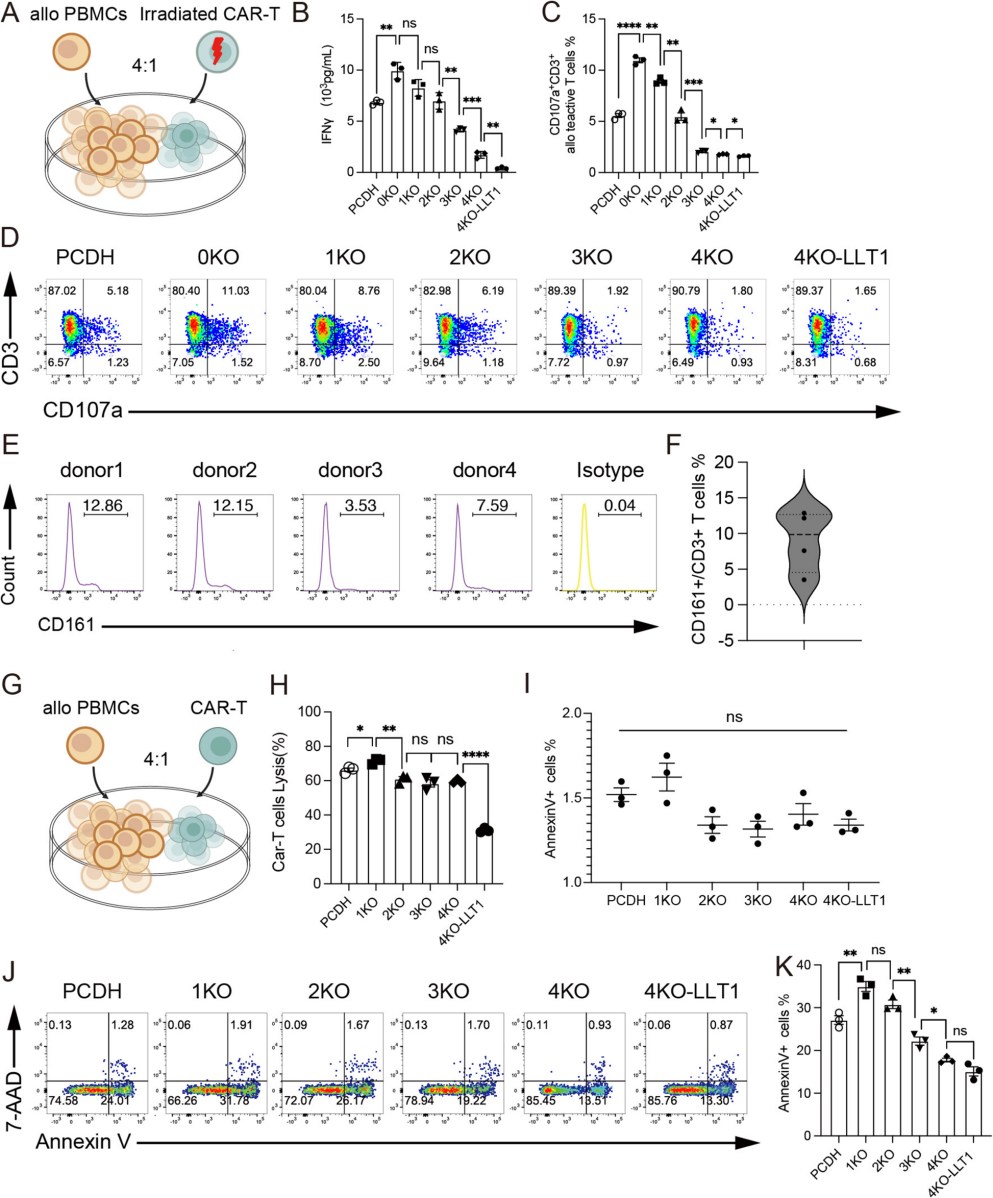
LLT1 overexpression renders resistance to allogenic PBMCs. **A** Schematic illustration of the (**B**) (**C**) (**D**) MLR (mixed lymphocyte experiment) (*n* = 3). Irradiated donor CAR-T cells were mixed with fourfold alloreactive PBMC cells. **B** The IFNγ concentration in supernatants of alloreactive PBMC cells stimulated by irradiated (30 Gy) CAR-T cells with different genes knocked out (*n* = 3). **C** Representative flow plots and (**D**) the histogram analysis illustrates the percentage of CD107a^+^CD3^+^ cells in alloreactive PBMCs, following a period of 48 h of stimulation by CAR-T cells (*n* = 3). **E** The flow plots illustrate the proportion of CD161 marker expression on the surface of T cells in the peripheral blood of four healthy donors. **F** The violin plot illustrates the distribution of data on the proportion of CD161 in(E) (*n* = 4). **G** Schematic illustration of the (**H**) (**I**) (**J**) MLR (mixed lymphocyte experiment) (*n* = 3). Viable donor CAR-T cells were mixed with fourfold alloreactive PBMC cells. **H** The diagram presents the cytotoxic activity of alloreactive PBMC cells target donor CAR-T cells by the proportion of lysed CAR-T cells (*n* = 3). **I** The five CAR-T cells and PCDH-T cells have similar basal line of apoptosis as measured by Annexin V staining antibody. The data represent the mean ± SEM (*n* = 4). ns, not significant (one-way ANOVA). **J** Representative flow plots and (K) The histogram analysis illustrates the percentage of Annexin V^+^ cells in CAR-T cells, following a period of 48 h of coculturing with alloreactive PBMC cells(*n* = 3). Data represent the mean ± SEM of triplicates. Statistical significance was determined by two-tailed unpaired Student's t-test, except noted by one-way ANOVA. *p < 0.05, **p < 0.01, ***p < 0.001, and ****p < 0.0001; ns, not significant

Silencing the B2M and HLA-I/II genes in universal CAR-T cells renders them resistant to allogeneic T cell attack. The HVGR, typically triggered by TCR-HLA mismatches in allogeneic T cells, can compromise CAR-T cells persistence in vivo. To investigate whether TCR- or HLA-deficient CAR-T cells can evade clearance by allogeneic T cells. We used fresh PBMCs from allogeneic donors, primed for 2 weeks with irradiated CAR-expressing T cells and IL-2 [32]. Two weeks after priming, the predominant cell types in the peripheral blood mononuclear cells (PBMCs) were a smaller proportion of B and NK cells, alongside an increased proportion of activated T cells (Fig.S8A) [33]. Monocytes and macrophages were absent due to the lack of GM-CSF. CAR-T cells were irradiated to prevent cytokine release and cytolytic activity against CD38^+^ cells and then subjected to a mixed lymphocyte reaction (MLR) with primed PBMCs (Fig. 6A). After 24 h of co-culture, IFN-γ concentration in the supernatant was measured by ELISA, and CD107a expression was assessed by flow cytometry to evaluate NK cell degranulation [33, 34]. The results indicated a progressive reduction in PBMC alloreactivity toward CAR-T cells, as evidenced by decreased IFN-γ levels and CD107a expression, with the greatest reduction observed in the 4KO-LLT1 CAR-T group (Fig. 6B-D). We propose that primed PBMCs are capable of recognizing antigens on CAR-T cells, but the combined modifications of reducing CD38, CD3, HLA-I, and HLA-II expression, along with LLT1 overexpression, collectively minimize alloreactivity. Among these modifications, HLA-I knockdown had the most significant effect on reducing alloreactivity. Additionally, PCDH-T cells may have less alloreactivity than 0KO and 1KO CAR-T cells since they do not have the 38BBz CAR antigen on their surface.

The expression of CD161 on the surface of primed PBMCs from four different donors was analyzed, revealing that, on average, 10% of T cells were CD161-positive (Fig. 6E-F). We hypothesized that the mechanism by which LLT1 overexpression reduces immunogenicity may be linked to CD161 expression on T cells. The cytotoxicity of primed PBMCs toward allogeneic CAR-T/PCDH-T cells was assessed within 24 h using cytotoxicity assays (Fig. 6G). The results showed that 1KO CAR-T and PCDH-T cells were lysed at a higher rate, whereas 2KO, 3KO, and 4KO CAR-T cells experienced lower lysis, with 4KO-LLT1 CAR-T cells showing the least lysis (Fig. 6H). Furthermore, we assessed the MLR's CAR-T/PCDH-T cells' apoptosis. The proportion of CAR-T cells with Annexin V^+^ decreased gradually from 1KO CAR-T to 4KO-LLT1 CAR-T cells (Fig. 6J-K), with no significant difference in the basal percentage of Annexin V^+^ CAR-T/PCDH-T cells (Fig. 6I).

To sum up, our results show that combining LLT1 overexpression with TCR and HLA gene knockdown dramatically lowers immunogenicity and increases CAR-T cell resistance to allogeneic T cell-mediated lysis.

### LLT1-overexpressing UCAR-T cells demonstrate potent anti-tumor activity in humanized T-ALL models

**Fig. 7:**
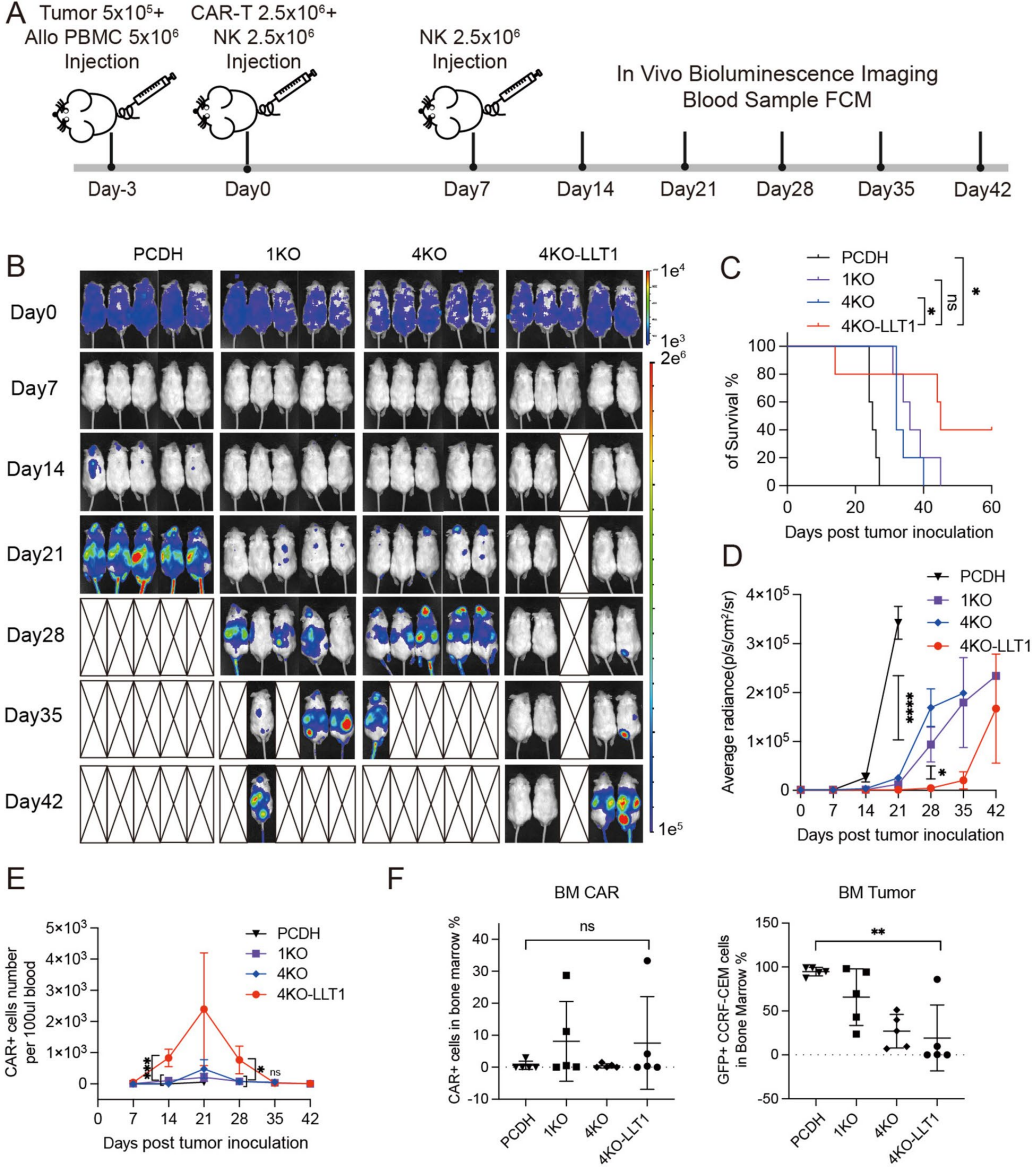
LLT1-overexpressing UCAR-T cells demonstrate potent anti-tumor activity in humanized T-ALL models. **A** Animal experimental timeline. Flowchart shows that mice (*n* = 5) were injected intravenously with 5 × 10^5^ CCRF-CEM-Luc-GFP-B2M^KO^ cells and 5 × 10^6^ HLA-A2 mismatched alloreactive PBMC cells, followed by 2.5 × 10^6^ CAR-T/PCDH-T cells and 2.5 × 10^6^ NK cells three days later. And same dose NK cells were re-injected after 7 days of CAR-T inoculation. Tumor burden of mice was measured every 1 week. **B** Bioluminescence imaging (BLI) results of tumor burden. **C** Survival curves of humanized T-ALL model with alloreactive PBMCs (*n* = 5), the statistical significance was determined with log-rank test (Mantel-Cox). **D** The mean value of average radiance in each group of mice was monitored using bioluminescence. **E** The plot shows the quantity of CAR-T cells per 100 µl of mouse peripheral blood. Flow cytometry was conducted once a week to identify HuCD45^+^ CAR^+^ cells within the peripheral blood of mice (*n* = 5). **F** The plots show the results of flow cytometry analysis of the percentage of CAR-T cells and tumor cells in bone marrow cells collected from each mouse at the time of death (*n* = 5), the statistical significance was determined with one-way ANOVA. All data are presented as mean ± SEM and analyzed by two-tailed unpaired Student's t-test, except noted by one-way ANOVA and log-rank test (Mantel-Cox). *p < 0.05, **p < 0.01, ***p < 0.001, and ****p < 0.0001; ns, not significant

Building on our in vitro findings, we used a humanized mouse model of T-ALL to assess the therapeutic effectiveness of 4KO-LLT1 CAR-T cells. NSG mice were intravenously injected with 5 × 10^5^ CCRF-CEM-Luc-GFP-B2M^KO^ cells and 5 × 10^6^ HLA-mismatched recipient PBMCs, which were primed with irradiated donor CAR-T cells [35]. Three days later, mice received 2.5 × 10^6^ PCDH-T cells or CAR-T cells, along with 2.5 × 10^6^ allogeneic NK cells [36]. To account for the short lifespan of NK cells compared to T and B cells [37], NK cells were administered a second time on day 7 after the initial injection (Fig. 7A). Peripheral blood was collected weekly to monitor HuCD45^+^ CAR-T cell counts via flow cytometry and tumor progression was tracked using bioluminescence imaging (Fig. 7B). Compared to PCDH-T cell therapy, all CAR-T cell therapies significantly prolonged survival in tumor-bearing mouse. Notably, mice who received 4KO-LLT1 CAR-T cell treatment had the best survival rates; two of them survived for up to 60 days without experiencing a tumor recurrence. (Fig. 7C). By day 21 post-treatment, the 4KO-LLT1 CAR-T cell-treated mice demonstrated near-complete tumor clearance (Fig. 7D).

On days 14 and 28, the peripheral blood of mice receiving 4KO-LLT1 CAR-T cells contained significantly more CAR-T cells than those treated with 4KO and 1KO CAR-T cells (Fig. 7E), indicating superior expansion of 4KO-LLT1 CAR-T cells compared to the other groups. Bone marrow analysis of mice in the terminal phase showed that the highest percentage of HuCD45^+^ CAR-T cells in the 4KO-LLT1 and 1KO groups, while the 4KO-LLT1 group had the lowest average percentage of GFP^+^ tumor cells (Fig. 7F).

Pathological evaluation of vital organs revealed that mice treated with 1KO CAR-T and PCDH-T cells exhibited signs of inflammatory responses, such as mast cell infiltration of the skin (red arrow), pulmonary acid exudation, and thickening of the alveolar walls (green arrow) (Fig.S8G). This suggests a potential immune-related inflammatory response in the skin and lungs during treatment. In contrast, treatment with 4KO CAR-T and 4KO-LLT1 CAR-T cells in humanized mice mitigated these inflammatory responses, which may be attributed to the deletion of TCR and HLA genes.

## Discussion

The goal of this study was to develop a universal CD38-targeted CAR-T cell that exhibits reduced immunogenic and resistance attacks from allogeneic NK and T cells, while maintaining their functionality in a homogeneous allogeneic environment. Using CRISPR/Cas9 technology in conjunction with lentiviral gene overexpression, we generated 4KO-LLT1 CAR-T cells, which are universal CD38-targeted CAR-T cells with low immunogenicity. 4KO-LLT1 CAR-T cells overexpressed LLT1 but lacked CD3, CD38, MHC1, and MHC2 expression. Their ability to evade attacks by allogeneic T and NK cells was verified through both in vitro and in vivo testing. Deletion of HLA-I and HLA-II naturally reduces the risk of rejection by allogeneic T cells due to HLA-TCR mismatches. In addition, LLT1 overexpression further inhibited allogeneic T cells response. Furthermore, LLT1 overexpression enhanced the cytokine profile and preserved the naive/stem-like characteristics of the CAR-T cells. These modifications led to improved survival and enhanced resistance to cancer in the in vivo models.

Several strategies can be employed to prevent NK cell-mediated lysis, in addition to universal CAR-T cells that overexpress LLT1 on their surface. According to Mo et al. [38], one strategy is to provide CAR-T cells with a lysing function that targets NK cells such as the alloimmune defense receptor (ADR). Other strategies involve expressing inactivating receptors of NK cell, such as B7H3 and CD155, on the CAR-T cells' surface [39], as well as members of the SLAM family of receptors (e.g., CD48, CD229) [40]. Additionally, CD47 molecules [41] have been shown to inhibit macrophage-mediated rejection, while conventional HLA-E/HLA-G molecules [42] can mediate anti-NK rejection effects. However, our results support the potential of overexpressing LLT1 as an effective means of protecting CAR-T cells from rejection by allogeneic NK and T cells. Future research will focus on exploring the therapeutic potential of LLT1, particularly its ability to prevent alloimmune responses in other tumor therapies beyond ALL.

Targeting a single NK pathway, such as LLT1, is limited by the intricate balance between the activating and inhibitory signals that regulate NK cell function. This strategy may not fully capture the diversity of NK responses in complex tumor microenvironments, where alternative signals may dominate and NK cells may adapt or develop resistance. To address this, combination therapies targeting multiple NK cell pathways offer a more comprehensive approach, enhancing efficacy and reducing the risk of immune escape. Such strategies could provide a robust solution for overcoming NK cell-mediated rejection in CAR-T therapies, thereby addressing the limitations of single-pathway targeting.

We conducted a comprehensive analysis of 4KO-LLT1 CAR-T cells, evaluating cytokine secretion, antigen-specific proliferation, immunophenotyping, and transcriptomic profiling. Our findings indicate that LLT1 overexpression enhances cytokine and cytokine receptor interactions, leading to a marked increase in IL-10 production. Importantly, further inhibition assays confirmed that the elevated IL-10 secretion is regulated by LLT1 through activation of the classical NF-kB pathway. This elevated IL-10 level likely contributes to the superior antitumor activity observed in 4KO-LLT1 CAR-T cells [43]. Moreover, LLT1 overexpression was found to modulate the differentiation of CAR-T cells, inhibiting their transition into effector phenotypes while promoting the expression of CD62L^+^CD45RA^+^ markers indicative of a naive phenotype. Transcriptomic profiling supports this observation, revealing enrichment in stemness-associated genes, including *BCL6*, *TCF7*, *CXCR3*, and *CD27*, which underscores a highly naive and stem-like cellular state. Notably, *TCF7*, as a nuclear transcription factor, was identified as a key regulator contributing to the enhanced naive and stem-like phenotype of 4KO-LLT1 CAR-T cells. These findings align with recent studies showing that CAR-T cells with overexpression of IL-10 exhibit memory-like properties similar to those of stem cells during tumor clearance [43]. Our findings align with this observation, as the stem-like phenotype of 4KO-LLT1 CAR-T cells, characterized by TN/TSCM populations, supports enhanced viability, robust antigen-specific proliferation, and the capacity to differentiate into diverse memory and effector T-cell subsets [44].

In addition to its crucial function in controlling immunological responses, CD38 is essential for cell activation, proliferation, and differentiation. Additionally, CD38 exhibits extracellular enzymatic activity, functioning as both a cyclase and hydrolase [45]. Recently, Yue et al. identified CD38 as a marker of CAR-T cell exhaustion using single-cell multiomics data analysis, with further validated in vitro models. Notably, CD38 knockdown suppresses HIF1A, a key regulator of glycolysis, through the CD38-NAD^+^ -SIRT1 pathway [46]. Moreover, CD38 knockdown has shown to enhance the functionality of CD19 CAR-T cells and improves tumor responsiveness [47]. Consistent with these findings, CD38 knockdown in our study may help inhibit CAR-T cell fratricide while simultaneously augmenting CAR-T cell antitumor activity. These results highlight the potential of targeting CD38 as a strategy to optimize CAR-T cell therapy outcomes.

CD38 is expressed at varying levels across different hematological malignancies but is generally absent in hematopoietic stem cells. For example, CD38 is found in 100% of multiple myeloma (MM) cases [48], 58.2% - 78.2% of acute leukemia cases [18], 30%−50% of chronic lymphocytic leukemia cases [49], and 50–80% of T-cell lymphomas [17]. Qingya et al. evaluated the efficacy and safety of CD38 CAR-T cell therapy in patients with relapsed acute myeloid leukemia (AML) after allogeneic hematopoietic stem cell transplantation (allo-HSCT) [19], reporting that all six patients achieved complete remission (CR) without off-target effects on monocytes and lymphocytes. Additionally, CD38^+^ CAR-T cells have shown promising efficacy for treating MM and acute lymphoblastic leukemia (ALL) [20]. Despite these advances, many hematological malignancies remain difficult to cure, with high recurrence rates and poor prognoses. The time-consuming and quality-variable process of CAR-T manufacturing presents additional challenges. Developing universal CD38 CAR-T cells could address these issues, enabling the cryopreservation of ready-to-use CAR-T products for widespread applications. Such a universal product could be employed as a stand-alone therapy or in combination with other immunotherapies such as stem cell transplantation and additional targeted CAR-T approaches.

When compared to autologous CAR-T, off-the-shelf CAR-T treatments for NHL and ALL may lower the incidence of immune effector cell-associated neurotoxicity syndrome (ICANS) and severe cytokine release syndrome (CRS) [50]. According to earlier research, off-the-shelf CAR-T cells do not overproliferate and produce CRS. This is probably because the patient's immune system rejects them because of HLA loss or mismatch, which makes them appear alien [51]. A case report on CD19 UCAR T-cell therapy highlighted that its efficacy in patients with B-cell ALL is limited to 28 days, with the brief persistence of UCAR T-cells being identified as a major factor contributing to relapse [7]. In this context, introduction of LLT1 offers a promising strategy for mitigating rejection by the alloimmune system, thereby enhancing the durability and effectiveness of CAR-T therapy. Furthermore, LLT1 overexpression helps maintain CAR-T cells in a stemness and naive state, potentially promoting prolonged persistence while reducing the risk of tumor overactivation, thus lowering the likelihood of severe CRS.

Most studies assessing the efficacy of CAR-T cell therapy have employed NSG mice to establish cell line-derived xenograft (CDXs) models [20, 32, 38]. These mice were genetically engineered to be Prkdc^scid^ or Rag1/2 deficient and lack the il2rg locus, making them immunodeficient. Although these models are invaluable for evaluating CAR-T cell activity against tumors, they lack a functional human immune system, limiting their utility in investigating the interactions between CAR-T cells and immune components. To address this limitation, we utilized humanized mouse models to better assess the efficacy of universal CAR-T cells in an immuno-oncology context. Initially, we employed the Hu-SRC-SCID model, which was generated via transplantation of CD34 ^+^ hematopoietic stem cells from the umbilical cord blood into immunodeficient mice. This model supports development of the human immune system [52]. However, significant limitations have been observed, including the inability to elicit realistic host rejection responses in CAR-T cells. Peripheral blood analysis revealed undetectable levels of T, B, and bone marrow cells within 4–8 weeks post-transplantation, prompting us to discontinue its use. Subsequently, we used the Hu-PBL-SCID model generated by injecting human peripheral blood mononuclear cells (PBMCs). This model enables rapid establishment of human CD3 ^+^ T cells and is particularly suited for studying T cell functionality in vivo [53]. Despite certain drawbacks such as a limited experimental window (4–8 weeks), graft-versus-host disease (GvHD) risks, and a low survival rate of NK cells [52], this model proved to be the most suitable for our investigation. Notably, we observed several advantages: (1) the experimental period aligned with the typical expansion duration of CAR-T cells (≤ 28 days); (2) NK cells could be supplemented via additional injections; (3) no GvHD symptoms (e.g., weight loss, hair loss) were observed; and (4) universal CAR-T cells demonstrated efficacy against rejection by allo-T cells and allo-NK cells. These findings underscore the potential of the Hu-PBL-SCID model as an effective platform for evaluating universal CAR-T-cell therapy in vivo.

Some limitations were identified in this work when the Hu-PBL-SCID model was used to assess the immunogenicity and antitumor effectiveness of universal CAR-T cells. This model lacks mature innate immune cell populations including monocytes, macrophages, plasma cells, and dendritic cells (DCs), which are essential for generating robust antigen-specific antibody responses. Consequently, it was difficult to discern any possible signs of graft-versus-host disease (GvHD) brought on by allogeneic CAR-T (such as 1KO CAR-T). Furthermore, preclinical research on 4KO-LLT1 CAR-T cells, which have been suggested as a novel universal CAR-T cell treatment, is still in its early phases. To confirm its therapeutic potential, thorough clinical trials is essential.

In conclusion, our findings indicate that LLT1 overexpression effectively mitigates allogeneic NK cell rejection. In humanized mouse models, anti-CD38 universal CAR-T cells demonstrated potent antitumor efficacy. As ready-to-use therapeutic products, they offer promising treatment options for various hematological malignancies. Notably, LLT1 overexpression stimulates the production of IL-10, a cytokine that may be crucial for preserving the stemness of CAR-T cells, in addition to improving the naïve phenotype of CAR-T cells. These results provide valuable insights to support the further development and practical use of universal CAR-T treatments.

## Conclusion

Our study highlights the transformative potential of allogeneic CD38-targeting universal CAR-T cell therapy for hematological malignancies. Through the integration of multiple innovative strategies, including the disruption of CD38, TCR, HLA-I, and HLA-II, and the overexpression of LLT1, we established a UCAR-T cell platform with significantly reduced immunogenicity and enhanced allogeneic persistence. Among these strategies, LLT1 plays a pivotal role, not only by shielding UCAR-T cells from allogenic NK cell-mediated rejection, but also by promoting their stemness, a critical feature that underpins durable anti-tumor efficacy, as demonstrated in both in vitro experiments and humanized models. These findings provide a solid foundation for future preclinical investigations and clinical trials to optimize this therapeutic approach. This UCAR-T-cell platform has enormous potential as an approachable and successful therapy option for patients with a range of hematological malignancies with further development and validation.

## Supplementary Information

Supplementary Material 1. Supplementary Fig. 1: (A) Representative flow plots illustrate the percentage of Annexin V^+^/7-AAD^+^ cells in CAR-T cells without stimulation, (B) The fold plots illustrate the expansion of 38BBz CAR-T cells with and without CD38 knockdown in T-cell medium with 100 ng/ml IL-2 and 10%FBS within 7 days. (C) The gating strategy for cell sorting and flow cytometry immunophenotyping is as follows: we first gated the main cell population based on FSC/SSC to exclude debris, then used FSCH/A to exclude doublets and select singlet cells. Next, we gated for viable cells by excluding DAPI-positive dead cells. Using PCDH-T cells as a positive control, we identified cells negative for CD3, CD38, MHC-1, and MHC-2 expression. Finally, within the negative population, we gated cells positive for CAR expression. For 4KO-LLT1 CAR-T cells, only CAR-positive cells were sorted due to co-expression of the CAR epitope and LLT1. The same strategy was applied for 1KO, 2KO, and 3KO CAR-T cells. Supplementary Fig. 2: (A) Flow cytometric plots showing proportion of CD4^+^ T cells and CD8^+^ T cells in CAR-T/PCDH-T cells in unstimulated and antigen-stimulated condition. (B) The histogram shows the proportion of CD4^+^ T cells and CD8^+^ T cells in CAR-T/PCDH-T cells(*n* = 4) and proportion of CD4^+^ T cells and CD8^+^ T cells in CAR-T/PCDH-T cells which were stimulated with CCRF-CEM cells at ratio of 1:1(*n* = 4). Supplementary Fig. 3: (A) Surface overexpression of CD38 in CCRF-CEM, Raji, Molm13, Molt4, and Nalm6 cell lines (red histograms) versus FITC isotype (blue histograms) measured by flow cytometry. (B) Surface expression of GFP in CCRF-CEM, Raji and Molm13 cell lines (red histograms) versus isotype controls (blue histograms) measured by flow cytometry. (C) Surface expression of LLT1 in CCRF-CEM, Raji and Molm13 cell lines (red histograms) versus isotype controls (blue histograms) measured by flow cytometry. (D) Flowchart shows that mice (*n* = 4) were injected intravenously with 3 × 10^5^ CCRF-CEM-Luc-GFP cells, followed by 2.5 × 10^6^ CAR-T/PCDH-T cells seven days later. Tumor burden of mice was measured every week. (E) Bioluminescence imaging (BLI) results of tumor burden. Control group refers to PCDH-T cells treated mice. (F) The mean value of average radiance in each group of mice was monitored using bioluminescence (*n* = 4). (G) Survival curves of NSG ALL model (*n* = 4), the statistical significance was determined with log-rank test (Mantel-Cox). (H) The plot shows the quantity of CAR-T cells per 100 µl of mouse peripheral blood. Flow cytometry was conducted once a week to identify HuCD45^+^ CAR ^+^ cells within the peripheral blood of mice (*n* = 4). Data are presented as mean ± SEM and analyzed by two-tailed unpaired Student's t-test. *p < 0.05, **p < 0.01, ***p < 0.001, and ****p < 0.0001; ns, not significant. Supplementary Fig. 4 (A) Flow plots show the memory phenotype of 38BBz-LLT1 and 38BBz CAR-T cells, TN, naive, CD62L^+^CD45RA^+^; CM, central memory, CD62L^+^CD45RA^−^; EM, effector memory, CD62L^−^CD45RA^−^; TE, terminal effector, CD62L^−^CD45RA^+^. (B) The histogram shows frequency of memory phenotypic proportions of 38BBz-LLT1 and 38BBz CAR-T cells (*n* = 3). (C) The graph illustrates the cytotoxic activity of 38BBz-LLT1 and 38BBz CAR-T cells after a 12-h period of co-culture with target cells at a range of E:T ratios (*n* = 3). (D) Representative flow plots and (E) The histogram analysis illustrates the percentage of CD107a^+^CD3^+^cells in alloreactive PBMCs, following a period of 48 h of stimulation by irradiated CAR-T cells (*n* = 4). (F) The plots present the proportion of lysed 38BBz-LLT1 and 38BBz CAR-T cells when autologous primary NK cells and CAR-T cells were co-cultured at different E:T ratios over a 24-h period (n = 3). Data are presented as mean ± SEM and analyzed by two-tailed unpaired Student's t-test. *p < 0.05, **p < 0.01, ***p < 0.001, and ****p < 0.0001; ns, not significant. Supplementary Fig. 5 (A) Heatmap of the differential clustering of the RNA-seq results for 4KO CAR-T and 4KO-LLT1 CAR-T before and after tumor infiltration. Differential gene entry criteria were FDR p value < 0.05 and [log2FoldChange] ≥|1|. (B) Representative flow plots illustrate the percentage of IL-10 expression in CAR-T cells with DMSO and 20 μmol JSH-23 in presence of CCRF-CEM. Supplementary Fig. 6: (A) The gating strategies and dot plot show the immunophenotyping of CD45^+^ CAR^+^ T cells from peripheral blood of mice models using flow cytometry. Data represent the mean ± SEM (*n* = 5). Supplementary Fig. 7: (A) Flow cytometric plots showing proportion of CD3^−^CD56^+^ NK cells of seven different donors after induced by IL-2 and IL-15 for a week. (B)&(C) The plots present the proportion of lysed UCAR-T cells (*n* = 3) when co-cultured with primary NK cells from different donors at different E:T ratios over a 24-h period. Donor in (B) with approximately 20% CD161 expression. (D) The plots present the proportion of lysed UCAR-T cells (*n* = 3) every 24-h when autologous primary NK cells and donor UCAR-T cells were co-cultured at different 1:1 ratio over a 72-h period. (E) Surface expression of CD107 in NK cells of three different donors measured by flow cytometry, when co-cultured with 4KO CAR-T and 4KO-LLT1 CAR-T (*n* = 3). (F) The histogram shows the ratio represents the level of CAR-T cells expansion (*n* = 3), when mixed and cultured with onefold allogeneic NK cells and 30-fold CCRF-CEM-Luc-GFP cells for 48 h. (G) Representative flow plots showing the percentage of CAR-T cells and tumor cells in the well plate at the two time points of day 0 and day 2. (H) The histogram shows the ratio of GFP-positive tumor cells lysed after 48 h of reaction in comparison to the control group, which comprised NK cells and tumor cells without CAR-T cells (*n* = 3). Data are presented as mean ± SEM and analyzed by two-tailed unpaired Student's t-test. *p < 0.05, **p < 0.01, ***p < 0.001, and ****p < 0.0001; ns, not significant. Supplementary Fig. 8: (A) The flow plots illustrate the proportion of T cells and NK cells in the primed PBMCs of four healthy donors. (B) Animal experimental timeline. Flowchart shows that mice (*n* = 5) were injected intravenously with 5 × 10^5^ CCRF-CEM-Luc-GFP-B2MKO cells and 5 × 10^6^ HLA-A2 mismatched alloreactive PBMC cells, followed by 2.5 × 10^6^ CAR-T/PCDH-T cells and 2.5 × 10^6^ NK cells 5 days later. Tumor burden of mice was measured every 1 week. (C) Bioluminescence imaging (BLI) results of tumor burden. (D) The mean value of average radiance in each group of mice was monitored using bioluminescence (*n* = 5). (E) Survival curves of humanized T-ALL model with alloreactive PBMCs (*n* = 5), the statistical significance was determined with log-rank test (Mantel-Cox). (F) The plot shows the quantity of CAR-T cells per 100 µl of mouse peripheral blood (*n* = 5). (G) Pathological analysis of the skin and lung of the representative four mice in every group at their terminal stage by HE staining. Magnification: 200 × . Data are presented as mean ± SEM and analyzed by two-tailed unpaired Student's t-test and log-rank test (Mantel-Cox). *p < 0.05, **p < 0.01, ***p < 0.001, and ****p < 0.0001; ns, not significant.

Supplementary Material 2. 

## Data Availability

The data generated and analyzed during this study are included in the article or can be accessed upon request from the corresponding authors.

## Abbreviations

ALL: Acute lymphoblastic leukemia
AML: Acute myeloid leukemia
B2M: Beta-2-microglobulin
CAR-T: Chimeric Antigen Receptor T-Cell Immunotherapy
CCR2: C–C Motif Chemokine Receptor 2
CIITA: Class II major histocompatibility complex transactivator
CRISPR/Cas9: Clustered regulatory interspaced short palindromic repeats/CRISPR-associated protein 9
CRS: Cytokine Release Syndrome
CXCR3: C-X-C motif chemokine receptor 3
FasL: Fas ligand
GVHD: Graft-versus-host disease
HLA: Human leukocyte antigen
HSCT: Hematopoietic stem cell transplantation
HVGR: Host-versus-graft reaction
ICANS: Immune effector cell-associated neurotoxicity syndrome
IFN-γ: Interferon gamma
Lag-3: Lymphocyte activating 3
LLT1: Lectin-like transcript 1
mAb: Monoclonal antibody
MHC: Major histocompatibility complex
MOI: Multiplicity of infection
NHL: Non-Hodgkin lymphoma
NK: Natural killer
PBMCs: Peripheral blood mononuclear cells
PD-1: Programmed cell death 1
RNP: Ribonucleoprotein
SELL: Selectin L
TCR: T cell receptor
TCF7: Transcription factor 7
Tim-3: Havcr2, hepatitis A virus cellular receptor 2
TIGIT: T cell immunoreceptor with Ig and ITIM domains
TNF-α: Tumor necrosis factor
TGFB3: Transforming growth factor beta 3
TRAC: T cell receptor alpha constant
UCAR-T: Universal chimeric antigen receptor-T cell
