# Supplementary material for: LLT1 overexpression renders allogeneic-NK resistance and facilitates the generation of enhanced universal CAR-T cells
Source: J Exp Clin Cancer Res. 2025 Jan 25;44:25. doi: 10.1186/s13046-025-03273-2 (PMC11763111; doi:10.1186/s13046-025-03273-2)
Supplement: Supplementary file 1 — Supplementary Material 1. Supplementary Fig. 1: (A) Representative flow plots illustrate the percentage of Annexin V+/7-AAD+ cells in CAR-T cells without stimulation, (B) The fold plots illustrate the expansion of 38BBz CAR-T cells with and without CD38 knockdown in T-cell medium with 100 ng/ml IL-2 and 10%FBS within 7 days. (C) The gating strategy for cell sorting and flow cytometry immunophenotyping is as follows: we first gated the main cell population based on FSC/SSC to exclude debris, then used FSCH/A to exclude doublets and select singlet cells. Next, we gated for viable cells by excluding DAPI-positive dead cells. Using PCDH-T cells as a positive control, we identified cells negative for CD3, CD38, MHC-1, and MHC-2 expression. Finally, within the negative population, we gated cells positive for CAR expression. For 4KO-LLT1 CAR-T cells, only CAR-positive cells were sorted due to co-expression of the CAR epitope and LLT1. The same strategy was applied for 1KO, 2KO, and 3KO CAR-T cells. Supplementary Fig. 2: (A) Flow cytometric plots showing proportion of CD4+ T cells and CD8+ T cells in CAR-T/PCDH-T cells in unstimulated and antigen-stimulated condition. (B) The histogram shows the proportion of CD4+ T cells and CD8+ T cells in CAR-T/PCDH-T cells(n = 4) and proportion of CD4+ T cells and CD8+ T cells in CAR-T/PCDH-T cells which were stimulated with CCRF-CEM cells at ratio of 1:1(n = 4). Supplementary Fig. 3: (A) Surface overexpression of CD38 in CCRF-CEM, Raji, Molm13, Molt4, and Nalm6 cell lines (red histograms) versus FITC isotype (blue histograms) measured by flow cytometry. (B) Surface expression of GFP in CCRF-CEM, Raji and Molm13 cell lines (red histograms) versus isotype controls (blue histograms) measured by flow cytometry. (C) Surface expression of LLT1 in CCRF-CEM, Raji and Molm13 cell lines (red histograms) versus isotype controls (blue histograms) measured by flow cytometry. (D) Flowchart shows that mice (n = 4) were injected intravenously with 3 × [file 13046_2025_3273_MOESM1_ESM.pdf]

Supplementary Table.1

Antibody Name	Supplier	Clone	Catalog
Percp-cy5.5 CD3	BioLegend	OKT3	317336
APC-cy7 CD3	BioLegend	HIT3a	300317
PE CD3	BioLegend	UCHT1	300407
FITC CD38	BioLegend	HB-7	356609
PE-cy7 CD38	BioLegend	HB-7	356608
PE HLA-A/B/C	BioLegend	W6/32	311405
Pe-cy7 HLA-DR/DP/DQ	BioLegend	Tu39	361707
Anti-hOCIL Affinity Purified Goat IgG	R&D		AF3480
AF488 donkey anti-goat IgG(H+L)	Invitrogen		2604365
CD38 Protein, Fc Tag	AcroBiosystems		CD8-H5255
APC Anti-Human IgG Fc	BioLegend	M1310G05	410712
PE CD4	BioLegend	A161A1	357404
APC CD8	BioLegend	SK1	344721
PE-cy7 Tim-3	BioLegend	F38-2E2	345013
Percp-cy5.5 Lag3	BioLegend	11C3C65	369311
PE PD-1	BioLegend	EH12.2H7	329905
APC TIGIT	BioLegend	A15153G	372706
BV510 CD45RA	BioLegend	HI100	304141
Pe-cy7 CD62L	BioLegend	DREG-56	304821
Pe-cy7 CD56	BioLegend	5.1H11	362509
Percp-cy5.5 CD161	BioLegend	HP-3G10	339908
APC CD107a	BioLegend	H4A3	328619
PE IL-5	BioLegend	TRFK5	504303
BV421 IL-10	BioLegend	JES3-9D7	501421
Pe-cy7IL-10	BioLegend	JES3-9D7	501419
BV605 IL-4	BioLegend	MP4-25D2	500827
Pe-cy7 CCR2	BioLegend	K036C2	357211
FITC CD45	BioLegend	HI30	304006
APC IgG2a, isotype	BioLegend	MOPC-173	400221

BV510 IgG1, isotype	BioLegend	MOPC-21	400171
PE IgG2a, isotype	BioLegend	MOPC-173	400213
FITC IgG2a, isotype	BioLegend	MOPC-21	400109
Pe-cy7 IgG2a, isotype	BioLegend	MOPC-173	400231
Percp-cy5.5 IgG2b, isotype	BioLegend	MPC-11	400337
BV421 IgG1, isotype	BioLegend	RTK2071	400429
BV605 IgG1, isotype	BioLegend	RTK2071	400433
PE anti-TCF1 (TCF7)	BioLegend	7F11A10	655207
Phospho-NF-κB p65 (Ser536) (PE Conjugate)	Cell Signaling Technology	93H1	5733S
