# Supplementary material for: LLT1 overexpression renders allogeneic-NK resistance and facilitates the generation of enhanced universal CAR-T cells
Source: J Exp Clin Cancer Res. 2025 Jan 25;44:25. doi: 10.1186/s13046-025-03273-2 (PMC11763111; doi:10.1186/s13046-025-03273-2)

Supplementary Figure1.

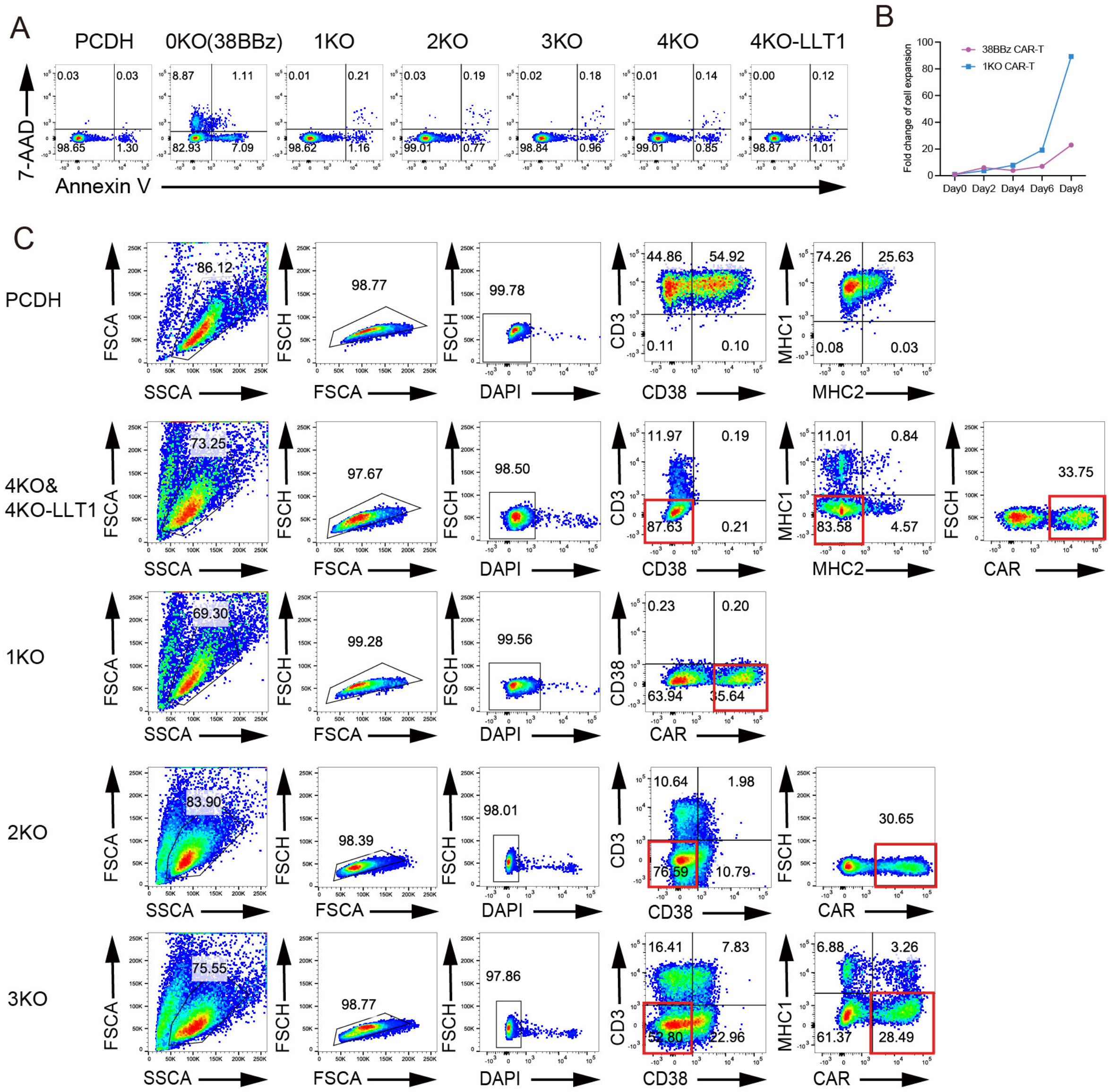

Supplementary Figure2.

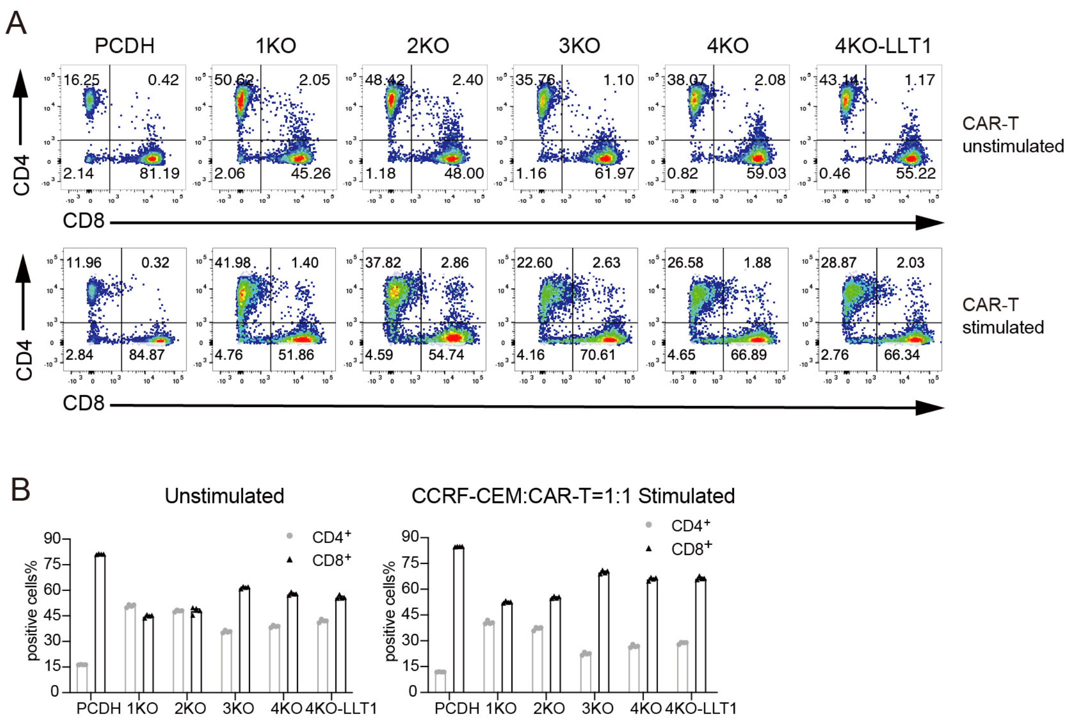

Supplementary Figure3.

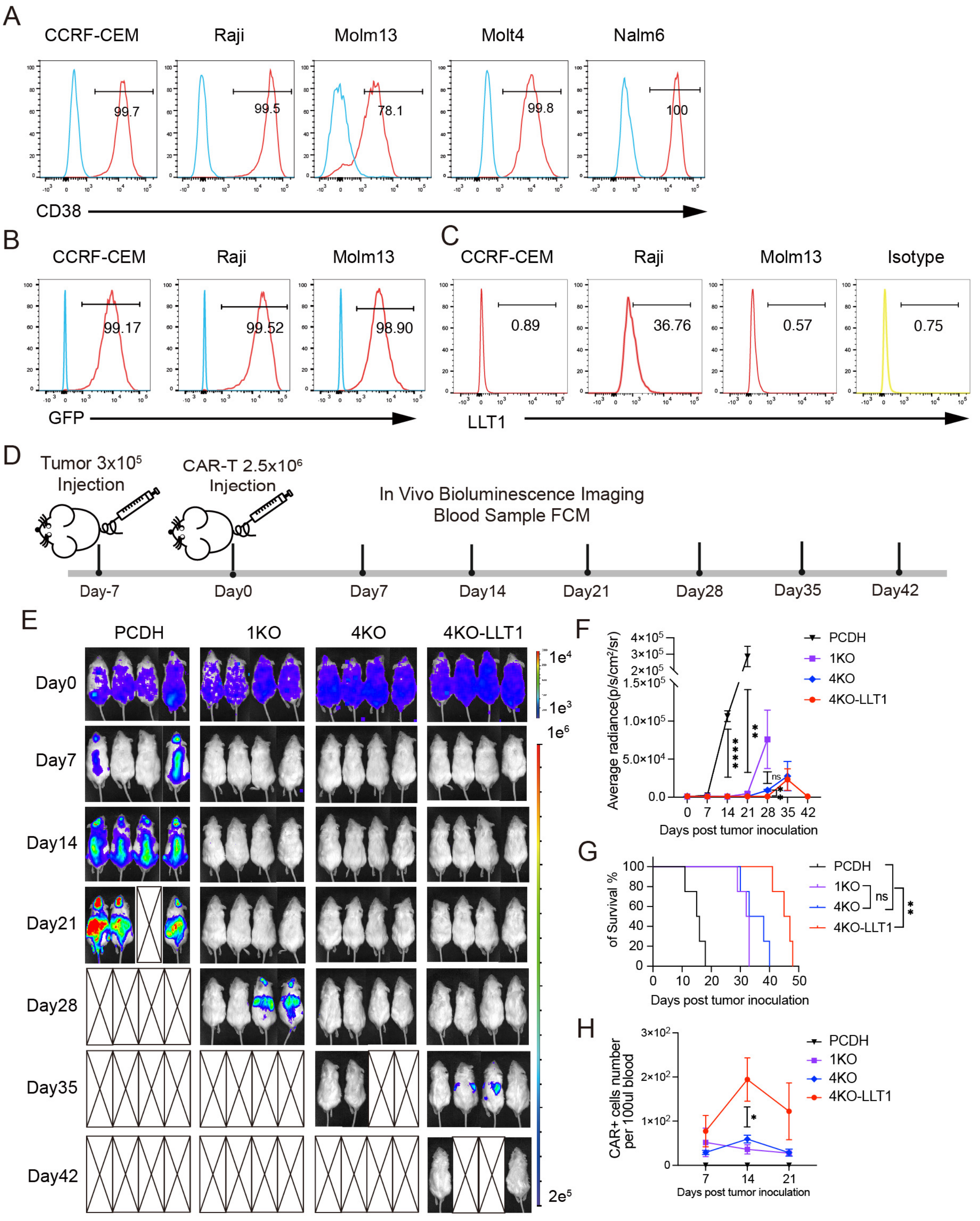

Supplementary Figure4.

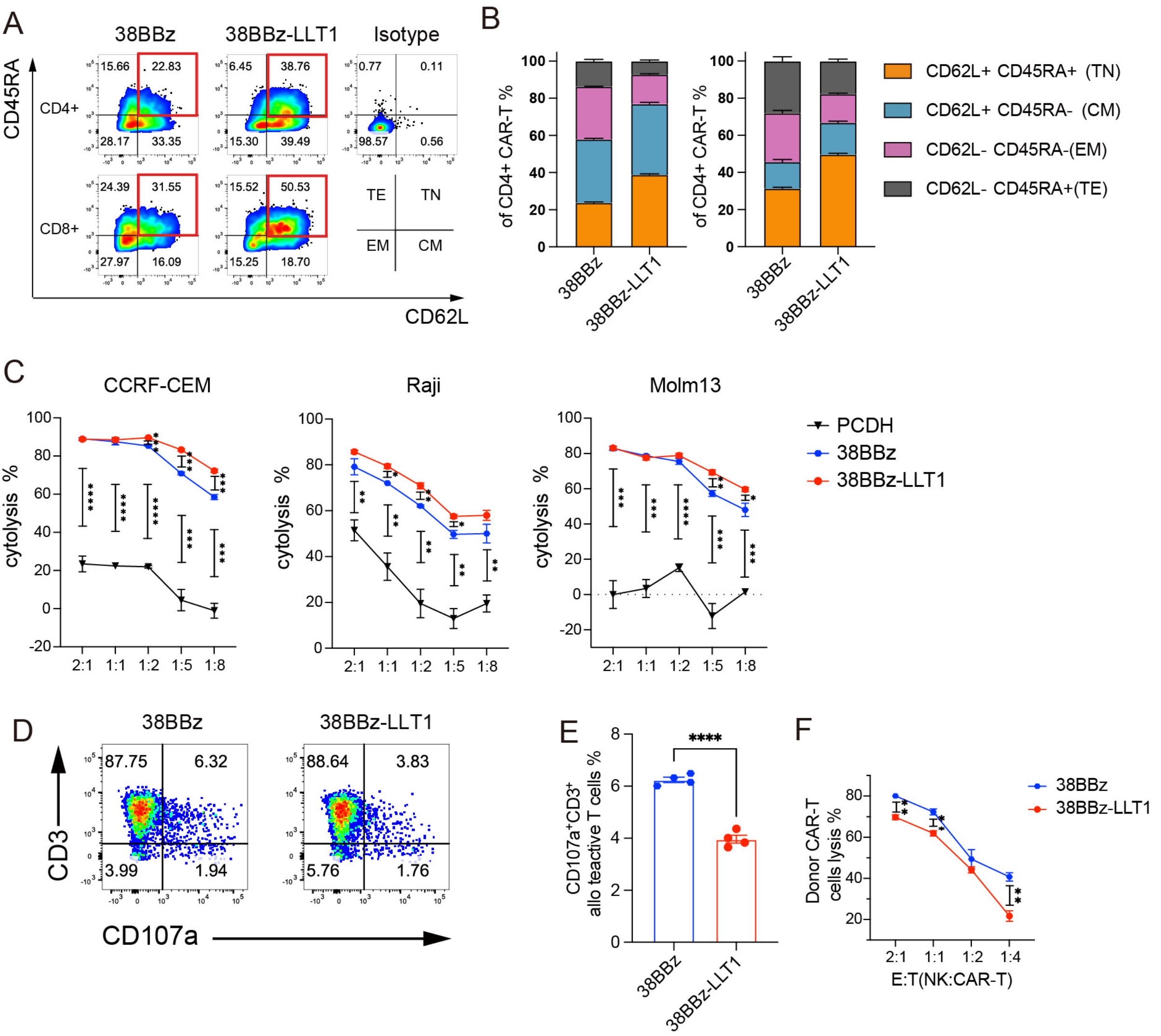

Supplementary Figure 5.

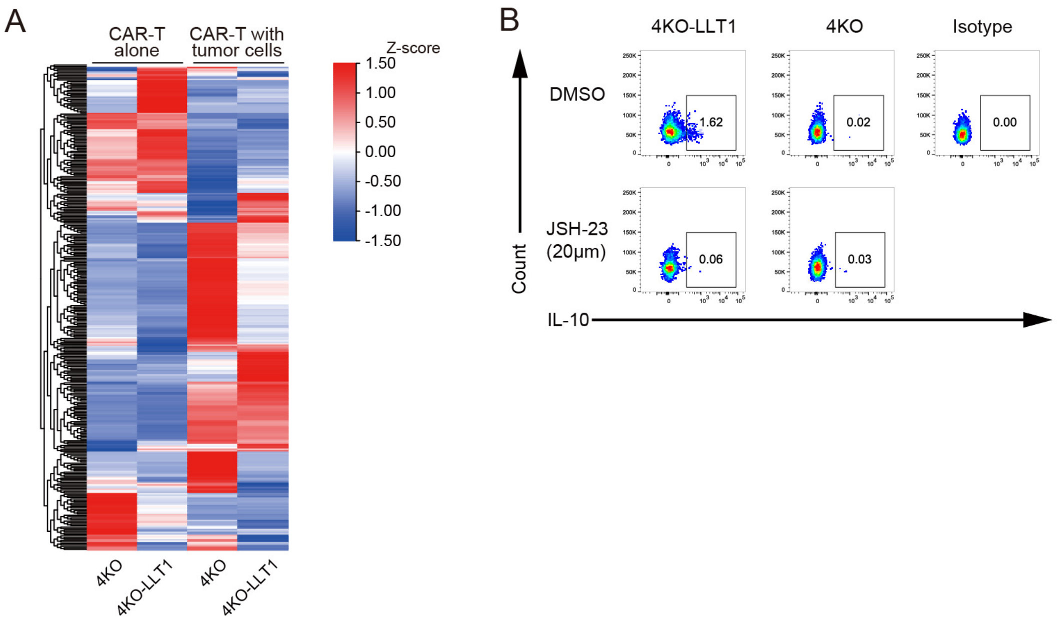

Supplementary Figure 6.

A

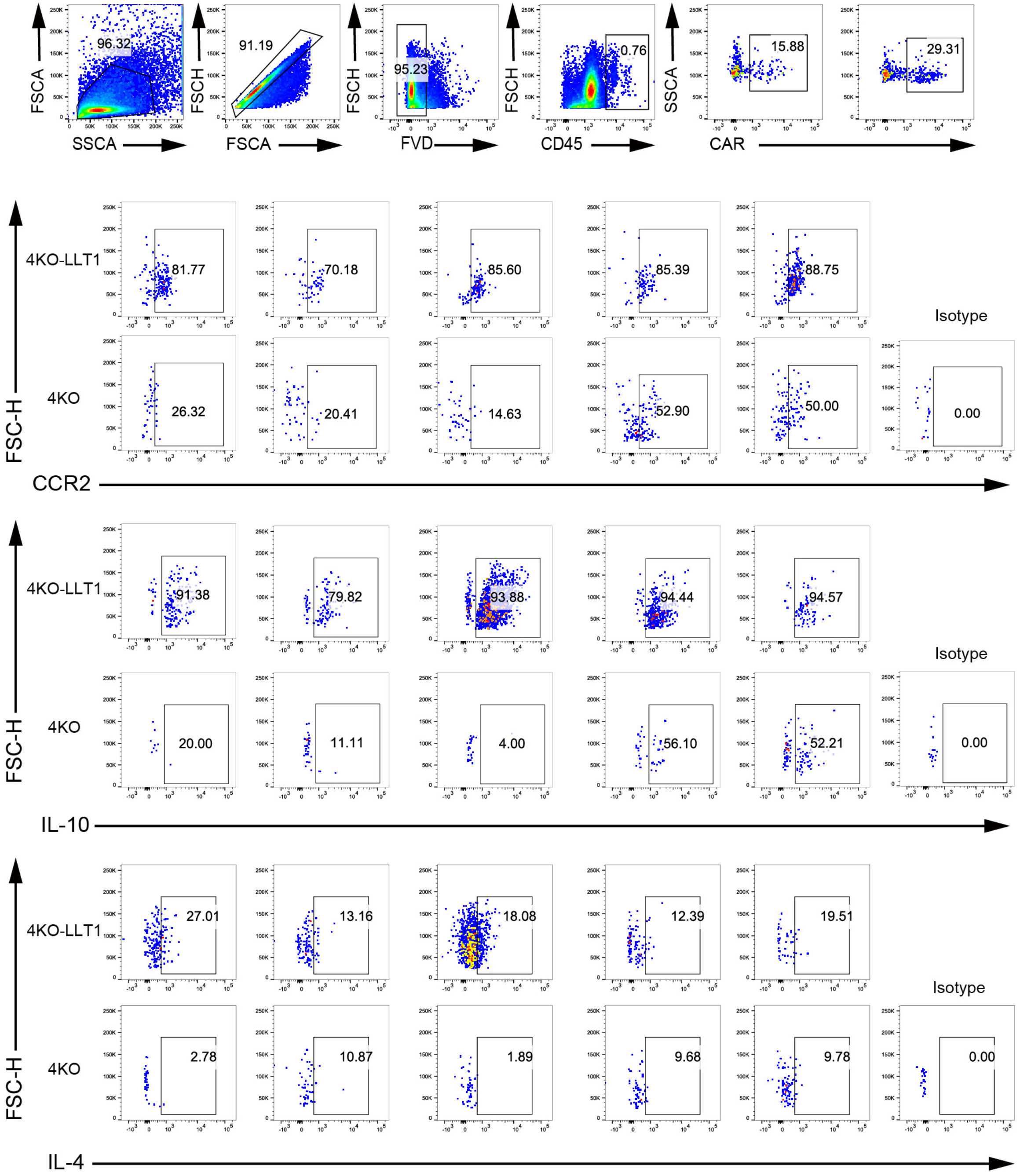

Supplementary Figure 7.

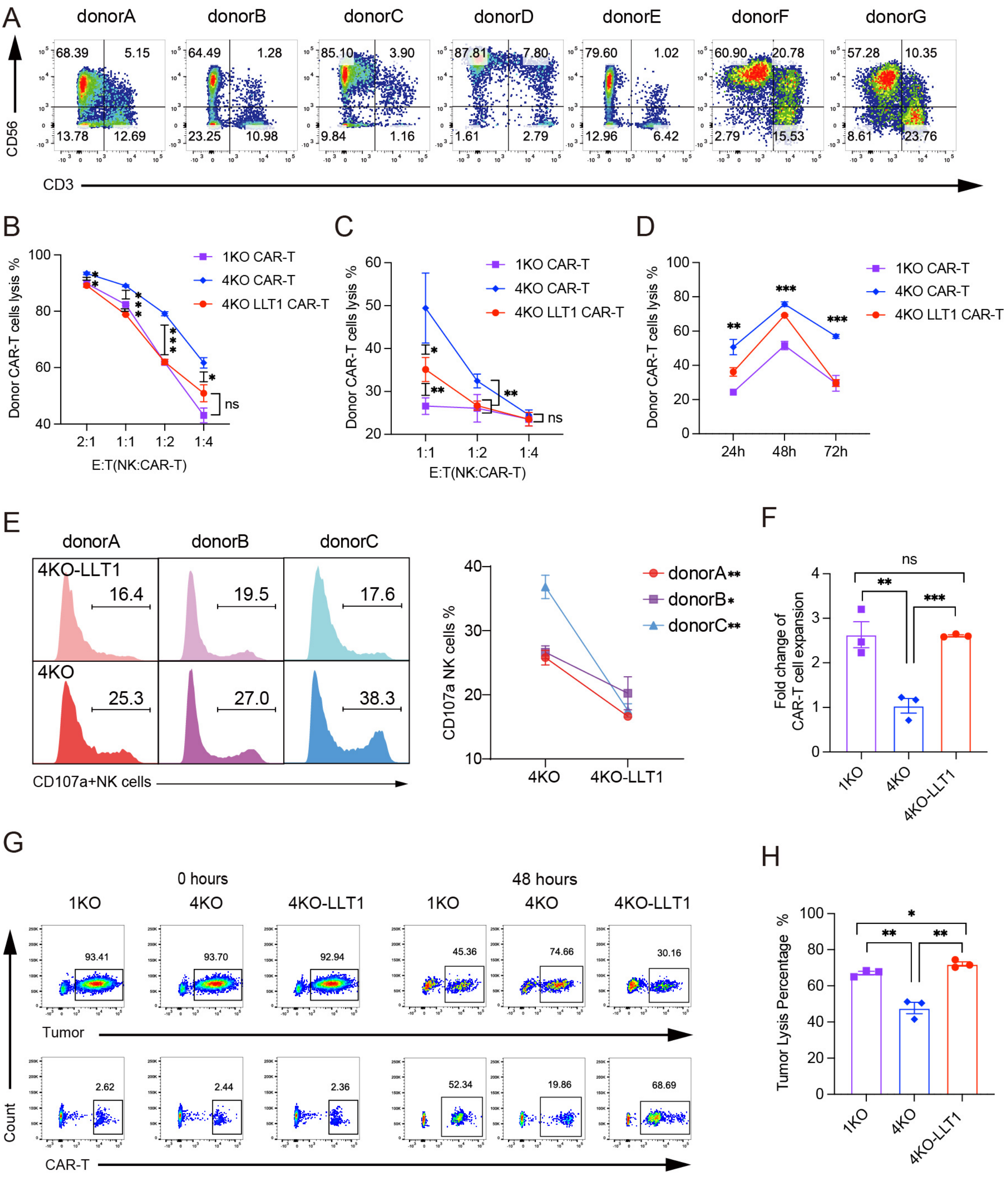

Supplementary Figure 8.

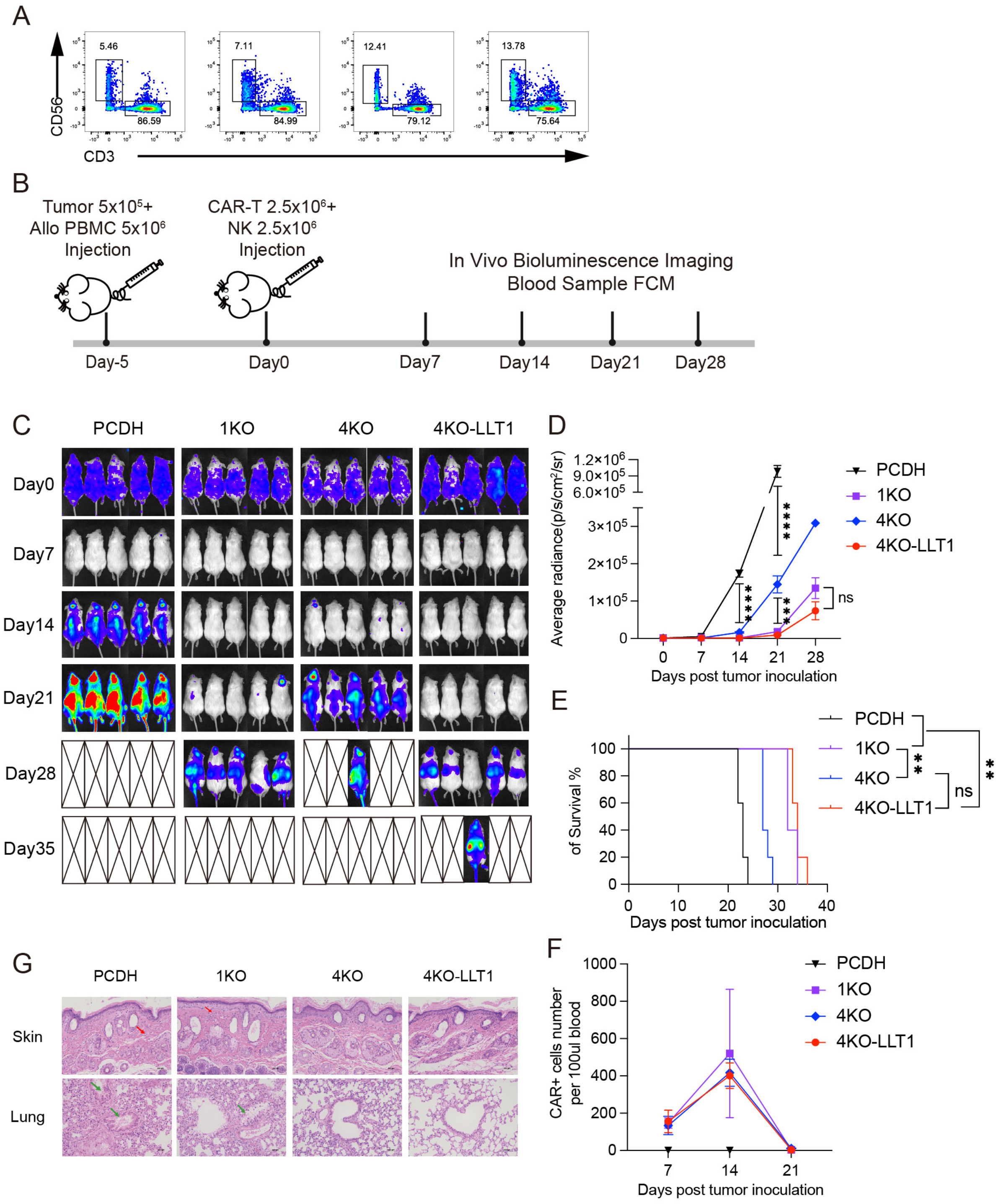

Supplement: Supplementary file 2 — Supplementary Material 2. [file 13046_2025_3273_MOESM2_ESM.pdf]
